# Functional Genomic and Biochemical Analysis Reveals Pleiotropic Effect of Congo Red on Aspergillus fumigatus

**DOI:** 10.1128/mBio.00863-21

**Published:** 2021-05-18

**Authors:** Zhonghua Liu, Shriya Raj, Norman van Rhijn, Marcin Fraczek, Jean-Philippe Michel, Odile Sismeiro, Rachel Legendre, Hugo Varet, Thierry Fontaine, Michael Bromley, Jean-Paul Latgé

**Affiliations:** a Unité des Aspergillus, Institut Pasteur, Paris, France; b Manchester Fungal Infection Group, The University of Manchester, Manchester, United Kingdom; c Institut Galien Paris Sud, UMR CNRS 8612, Châtenay-Malabry, France; d Transcriptome and Epigenome Platform, Biomics, C2RT, Institut Pasteur, Paris, France; e Bioinformatics and Biostatistics Hub, Department of Computational Biology, Institut Pasteur, Paris, France; Duke University Medical Center

**Keywords:** *Aspergillus*, antifungals, Congo red, galactosaminogalactan, germination

## Abstract

Inhibition of fungal growth by Congo red (CR) has been putatively associated with specific binding to β-1,3-glucans, which blocks cell wall polysaccharide synthesis. In this study, we searched for transcription factors (TFs) that regulate the response to CR and interrogated their regulon. During the investigation of the susceptibility to CR of the TF mutant library, several CR-resistant and -hypersensitive mutants were discovered and further studied. Abnormal distorted swollen conidia called Quasimodo cells were seen in the presence of CR. Quasimodo cells in the resistant mutants were larger than the ones in the sensitive and parental strains; consequently, the conidia of the resistant mutants absorbed more CR than the germinating conidia of the sensitive or parental strains. Accordingly, this higher absorption rate by Quasimodo cells resulted in the removal of CR from the culture medium, allowing a subset of conidia to germinate and grow. In contrast, all resting conidia of the sensitive mutants and the parental strain were killed. This result indicated that the heterogeneity of the conidial population is essential to promote the survival of Aspergillus fumigatus in the presence of CR. Moreover, amorphous surface cell wall polysaccharides such as galactosaminogalactan control the influx of CR inside the cells and, accordingly, resistance to the drug. Finally, long-term incubation with CR led to the discovery of a new CR-induced growth effect, called drug-induced growth stimulation (DIGS), since the growth of one of them could be stimulated after recovery from CR stress.

## INTRODUCTION

Congo red (CR) is an azo dye that was generally used in coloring treatment for textiles. However, due to its carcinogenic properties, its use has been limited. In histological examinations, CR is used for the identification of amyloidosis and the staining of several microorganisms (1). It has been known for a long time that CR can inhibit the growth of yeast and filamentous fungi. This drug binds to β-1,3-glucans, disorganizes the fungal cell wall structure due to inhibition of the crystallization of cell wall polymers, and affects cell wall polysaccharide synthesis (2–6). Accordingly, several components of the cell wall integrity and mitogen-activated protein kinase (MAPK) pathways are crucial for CR adaptation (7–10). Even though CR activity is clearly linked to the architecture of the cell wall, phenotypic screening of a collection of Saccharomyces cerevisiae null mutants has shown that the sensitivity of the mutants to CR differs markedly from that seen when exposed to other agents that disrupt cell wall biosynthesis, such as caspofungin (caspo), calcofluor white (CFW), and Zymolyase, indicating that CR activity is not limited to the inhibition of cell wall biosynthesis. In S. cerevisiae, CR hypersensitivity was associated with motor proteins and cytoskeletal organization, chromatin modification, or ubiquitination-related processes (2).

CR is active against filamentous fungi and especially the human pathogen Aspergillus fumigatus. As in S. cerevisiae, CR has an effect on cell wall mutants of A. fumigatus. For example, in this species, α-1,3-glucan synthase mutants are more sensitive to CR, whereas chitin synthase mutants, which have enhanced levels of glucans in their cell wall, are more resistant to CR. An A. fumigatus strain lacking β-1,3-glucan, although poorly growing, does not show different susceptibility to CR (11–14). Membrane transport and cell wall permeability may also play a part in CR activity in this fungus as there is a role for the hydrophobin RodA and two ABC transporters, AbcF and AbcH, in CR susceptibility (15, 16). A functional genomic study of Congo red activity in S. cerevisiae has allowed the identification of CRH (Congo red-hypersensitive) genes controlling susceptibility to Congo red (2). These genes do not, however, seem to play similar roles in filamentous fungi (17, 18). Taken together, these data suggest that the effects of CR are not exclusively due to inhibition of structural cell wall polysaccharides and could involve multiple pathways (19–21). Because of its wide use in fungal biology to interrogate cell wall biosynthesis genes, it is essential that the mechanism of CR is more clearly defined.

Interrogation of mutant libraries to identify strains that have altered sensitivity to antifungals has been highly effective in revealing mechanisms of drug action and resistance. Recently, a genome-wide transcription factor (TF)-null mutant collection has been generated in A. fumigatus (22). Screening the global TF-null mutant library for drug sensitivity can be used as a near proxy for a genome-wide screen if the assumption is made that the global transcription factor cohort is responsible for regulating the expression of the majority of the genes in the genome. A number of TF-null mutants have already revealed intriguing insights into CR action. RlmA, which is a member of the MADS (Mcm1-Agamous-Deficiens-serum response factor) box transcription factor family, functions as a regulator of the A. fumigatus cell wall integrity (CWI) pathway (23). ZipD, a basic leucine zipper transcription factor, regulates genes involved in calcium homeostasis (24). Δ*rlmA* and Δ*zipD* mutants are more sensitive to CR than their parental strain. In contrast, the loss of function of CrzA, a zinc finger transcription factor controlling calcium metabolism, leads to decreased susceptibility to CR (25, 26).

In this study, we have performed a series of functional genomic, phenotypic, and biochemical assays and reveal that A. fumigatus employs numerous strategies to overcome CR action, including modulating central metabolism, membrane and cell wall composition, and drug transport. In addition, we reveal that heterogenic conidial populations are able to mediate persistence in and detoxification of CR-containing environments and can be primed with the stress that they have been subjected to, leading to stimulation of growth.

## RESULTS

### Exposure to Congo red modifies the expression of multiple and diverse networks of genes in A. fumigatus.

**FIG 1:**
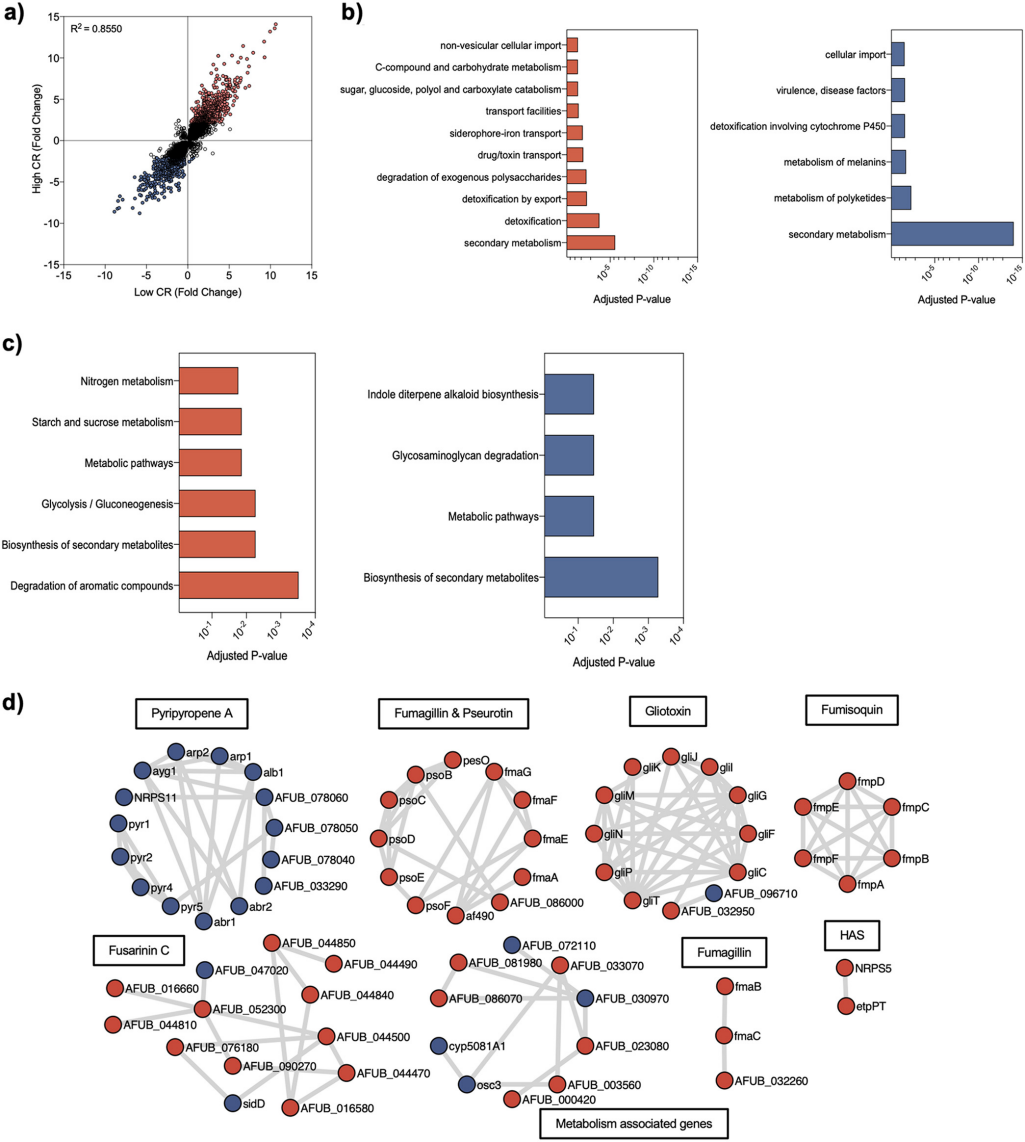
Transcriptome analysis of A. fumigatus MFIG001 under conditions of low (50 μg/ml) and high (300 μg/ml) concentrations of Congo red. (a) Correlation of gene expression of *P* value-filtered genes (*P*_adj_ < 0.05) under both conditions. (b) Significant FunCat terms of genes >2-fold upregulated (in red) or downregulated (in blue). (c) KEGG pathways enriched for genes differentially regulated under Congo red exposure. (d) STRINGS analysis of genes significantly up- and downregulated (>2-fold) (>50-base mean and *P*_adj_ of <0.05). HAS, hexadehydroastechrome. Upregulated genes are in red, and downregulated genes are in blue.

The antifungal properties of CR are thought to result predominantly, but not exclusively, from its ability to bind fibrillar chains of β-1,3-glucans, resulting in disruption of the cell wall structure. We hypothesized that an analysis of gene expression during exposure to Congo red, which has not been undertaken to date, would enable better characterization of the antifungal properties of this molecule. Two different concentrations, 50 μg/ml (low) and 300 μg/ml (high), of CR were added to actively growing mycelial cultures of A. fumigatus MFIG001 (27) and incubated for 4 h before harvesting. A total of 432 genes were upregulated and 271 genes were downregulated under low-CR conditions, while 656 genes were upregulated and 442 genes were downregulated under high-CR conditions (>2-fold; false discovery rate [FDR] of >0.05). Direct comparison of data sets from both low- and high-CR treatments revealed a high degree of correlation (*R*^2^ = 0.855), suggesting that increases in drug levels had little effect on those genes differentially regulated. However, transcriptional changes were enhanced with higher drug levels (Fig. 1a). Gene ontology (GO) analysis of the cohort of upregulated genes upon exposure to high CR concentrations revealed enrichment of categories linked to primary and secondary metabolism, detoxification, and transport (Fig. 1b and c). Analysis of the cohort of genes downregulated after CR treatment also showed a significant impact on genes involved in secondary metabolite biosynthesis. Analysis of protein networks using STRINGS allowed us to add further granularity to our analysis. The secondary metabolite pathways that were upregulated included those for the biosynthesis of gliotoxin, pseurotin, fumipyrrole, fumagillin, and siderophores. In contrast, all the genes of the cluster coding for the biosynthesis of the production of pyripyropene A were downregulated (Fig. 1d).

Our STRINGS analysis also revealed that a series of genes related to iron acquisition were upregulated in the presence of CR, specifically those for the production and uptake of the siderophores triacetylfusarinine C (TAFC) and ferricrocin (FC) (see Fig. S3 in the supplemental material). Interestingly, despite previously published work suggesting links between calcium metabolism and CR action (28, 29), the expression levels of genes associated with the calcineurin and calmodulin pathways, *calA*, *cnaB*, *cbpA*, *pmr1*, and *fbp1*, and other putative calcium binding proteins, *afua_1g02930* or *afua_1g0890*, were not modified in the parental strain in the presence of CR (https://doi.org/10.48420/14252756). Interestingly, genes upregulated in primary metabolism pathways included a number of glycosyl hydrolases that are involved in the sequestration of carbon from arabinan, xylan, xyloglucan, pectin, and cellulose (Fig. S4a). This transcriptional signature suggests that upon exposure to CR, A. fumigatus is reacting in a way consistent with carbon deprivation, specifically by upregulating genes used to degrade plant material in its natural environment in the soil. Although starvation responses in filamentous fungi are usually also associated with the upregulation of genes linked to conidiophore formation, we have observed a strong downregulation of *brlA*, *abaA*, *wetA*, *stuA*, and *mybA* TFs and genes controlling dihydroxynaphthalene-melanin and hydrophobin biosynthesis (https://doi.org/10.48420/14252756). This could be related to the fact that, in these experiments, the fungus is grown in liquid culture, where it does not normally conidiate.

10.1128/mBio.00863-21.3 FIG S3 Iron is not involved in the degree of resistance of strains to CR (a) Heat map of the genes involved in iron metabolism in the parental strain and the 4 TF mutants. (b) In the presence of iron starvation induced by the specific chelator BPS or after supplementation with a high concentration of iron (FeSO_4_), the sensitivity of strains to CR was not modified. Download FIG S3, PDF file, 0.4 MB. Copyright © 2021 Liu et al. 2021 Liu et al. https://creativecommons.org/licenses/by/4.0/ This content is distributed under the terms of the Creative Commons Attribution 4.0 International license.

10.1128/mBio.00863-21.4 FIG S4 Glycosyl hydrolase and cell wall genes. Shown are the expression levels of the glycosyl hydrolase genes involved in the degradation of plant decaying material (a) and cell wall-associated genes (b) by the parental strain and TF mutants. Download FIG S4, PDF file, 0.2 MB. Copyright © 2021 Liu et al. 2021 Liu et al. https://creativecommons.org/licenses/by/4.0/ This content is distributed under the terms of the Creative Commons Attribution 4.0 International license.

Since the function of CR has been associated with the disorganization of the cell wall, we expected to see differential expression of genes involved in cell wall biosynthesis and organization. As these genes are poorly annotated in STRINGS, we assessed these pathways directly (Fig. S4b). Contrary to our initial expectations, the expression levels of genes involved in the synthesis of β-1,3-glucans, chitin, or the UDP glucose pyrophosphorylase were unchanged. However, we observed some evidence of cell wall remodeling. Two members of the family involved in the reorganization of β-1,3-glucans, *Gel7* (AFUB_078410) and the essential gene *Gel4* (AFUB_022370), were overexpressed (30). The *ags1* (AFUB_014990) gene, which is the major driver of α-1,3-glucan synthesis (31), was overexpressed, while three α-1,3-glucanases were downregulated. The expression levels of genes of the UDP GlcNAc (*N*-acetylglucosamine), UDPGal, and UDP GalNAc synthetic pathways were also upregulated (32), suggesting that the supply of the appropriate substrate for chitin and galactosaminogalactan (GAG) synthesis was stimulated. In contrast, the expression of the UDP glucose pyrophosphorylase was unchanged. These data suggested that that the synthesis of the amorphous polysaccharides (α-1,3-glucans and galactosaminogalactan) was activated by the presence of CR.

### Screening of the TF-null mutant library revealed key transcriptional regulators that govern sensitivity to CR.

**FIG 2:**
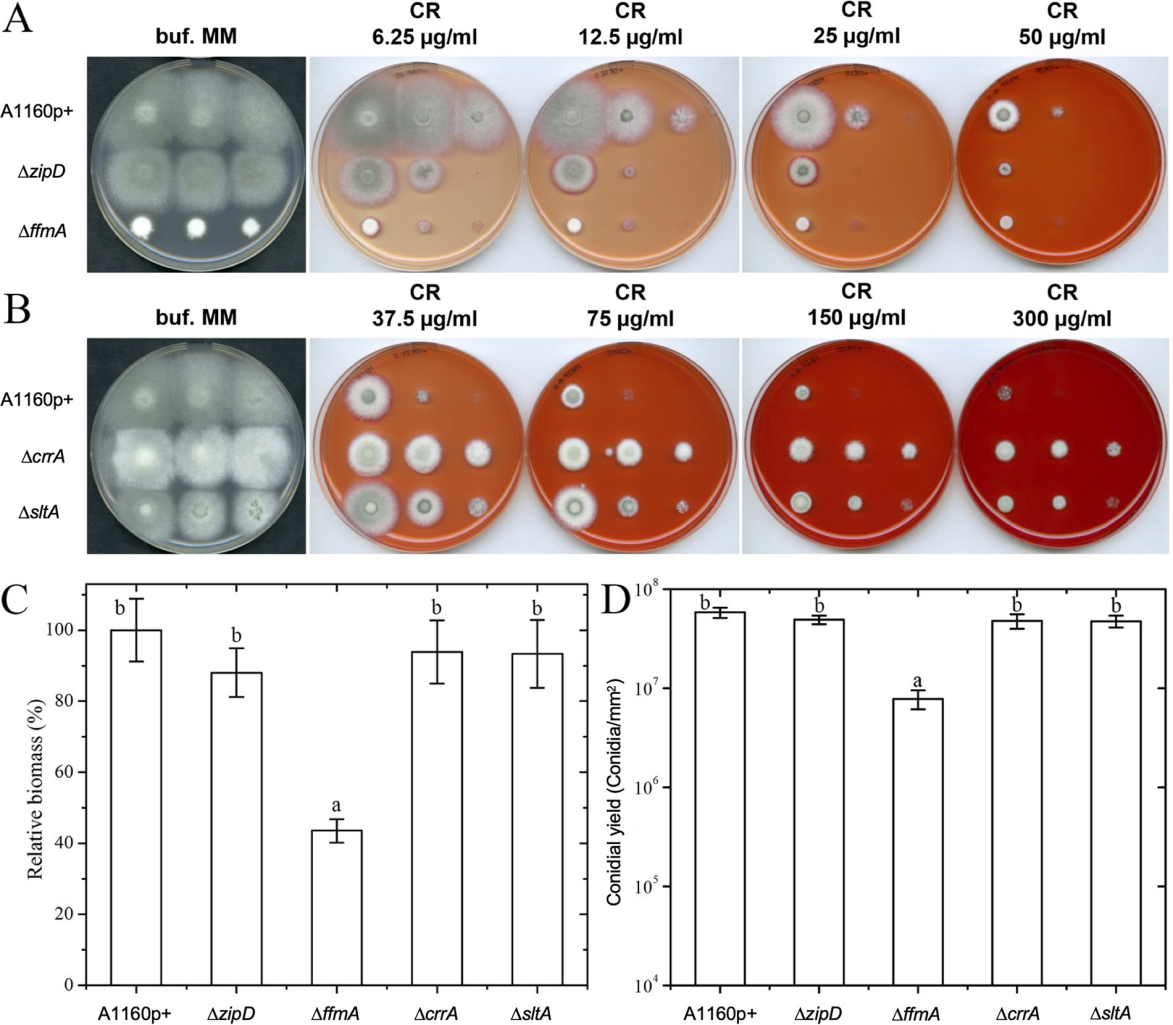
Susceptibility of MFIG001 and the Δ*zipD*, Δ*ffmA*, Δ*crrA*, and Δ*sltA* transcription factor mutants to Congo red. (A and B) The susceptibility of mutants was analyzed on a plate with different concentrations of CR to assess sensitivity (A) or resistance (B). (C and D) The relative biomasses of the wild type and mutants (C) and the conidial yields of these strains (D) were quantified in the absence of CR. The values represent the means ± standard deviations (SD) (*n* = 9). The biomass of the Δ*ku80* strain was set as 100%. Values marked with different letters are significantly different (*P* < 0.05).

To investigate regulatory pathways linked to CR responses, a comprehensive evaluation of regulators governing CR susceptibility was performed by screening the TF-null mutant library. The isogenic parental strain MFIG001 and mutants from the library were assessed on buffered minimal medium (MM) plates containing different concentrations of CR. Twelve mutants were identified as being more sensitive to CR than the parental strain, whereas six mutants were more resistant (Table 1). Consistent with previously published data, mutant 2C5, which lacks *rlmA* (AFUB_040580/AFUA_3G08520), and the 1B4 mutant, which lacks the gene that encodes the calcineurin pathway transcriptional activator *crzA* (AFUB_007280/AFUA_1G06900), were sensitive and resistant, respectively, to CR (25, 33). This result provided positive validation of our screen. Our results indicated the strains with the most dramatic phenotypes were isolates 1F4 (Δ*zipD*), 1G9 (here named Δ*ffmA*), and Δ*rlmA*, which had significant hypersensitivity growth defects at 12.5 μg/ml CR (Fig. 2A), while the most resistant isolates were Δ*crzA*, 1D4 (uncharacterized C6 transcription factor called CrrA, for Congo red resistant, here), 1G4 (annotated as RNA polymerase II transcription factor SIII elongin subunit A), and 2C7 (Δ*sltA*), all of which, unlike the parental strain, were able to grow with 300 μg/ml CR (Fig. 2B and Table 1). In the absence of CR, the growth rate and the conidial yield of the Δ*zipD*, Δ*crrA*, and Δ*sltA* mutants were indistinguishable from those of the parental isolate at 37°C in buffered MM (Fig. 2C). In contrast, the radial growth of the Δ*ffmA* mutant was reduced on solid MM, and the biomass recovered from liquid MM was reduced by 56%. Furthermore, the Δ*ffmA* mutant had a defect in the production of asexual spores, yielding <10% of that produced by the parental isolate (Fig. 2D).

**TABLE 1:** Identification of the transcription factor mutants with differential sensitivity to Congo red

Strain	Gene ID	Description	CR sensitivity^*a*^
1B4 (Crz1)	AFUB_007280	C_2_H_2_ transcription factor	+ +
1B8	AFUB_009970	CBF/NF-Y family transcription factor	−
1C3	AFUB_011800	Jumonji family transcription factor	−
1D4 (CrrA)	AFUB_014780	C6 transcription factor	+ + + +
1F4 (ZipD)	AFUB_020350	bZIP transcription factor	− − − −
1G11 (CreA)	AFUB_027530	C_2_H_2_ transcription factor	− −
1G4 (CrrB)	AFUB_024170	Low homology with RNA polymerase II transcription factor SIII (elongin) subunit A	+ + +
1G9 (FfmA)	AFUB_026340	C_2_H_2_ transcription factor	− − − −
2B3	AFUB_037150	PHD transcription factor	− −
2B5 (PacC)	AFUB_037210	C_2_H_2_ transcription factor	−
2C4	AFUB_040000	C6 transcription factor	+
2C5 (RlmA)	AFUB_040580	SRF-type transcription factor	− − −
2C7 (Ace1)	AFUB_041100	C_2_H_2_ transcription factor	+ + + +
2G7 (SteA)	AFUB_053740	Sexual development transcription factor	−
5B5	AFUB_024050	C6 finger domain protein	−
5C1	AFUB_038600	C6 finger domain protein	+
5H9 (MtfA)	AFUB_095620	C_2_H_2_ finger domain protein	−
6B1	AFUB_050260	MYB DNA binding domain protein	−

a +, more resistant than the parental strain; −, more sensitive than the parental strain; SRF, serum response factor; PHD, plant homeodomain.

### Transcriptomic evaluation identifies a role for FfmA in regulation of bioenergetic homeostasis.

**FIG 3:**
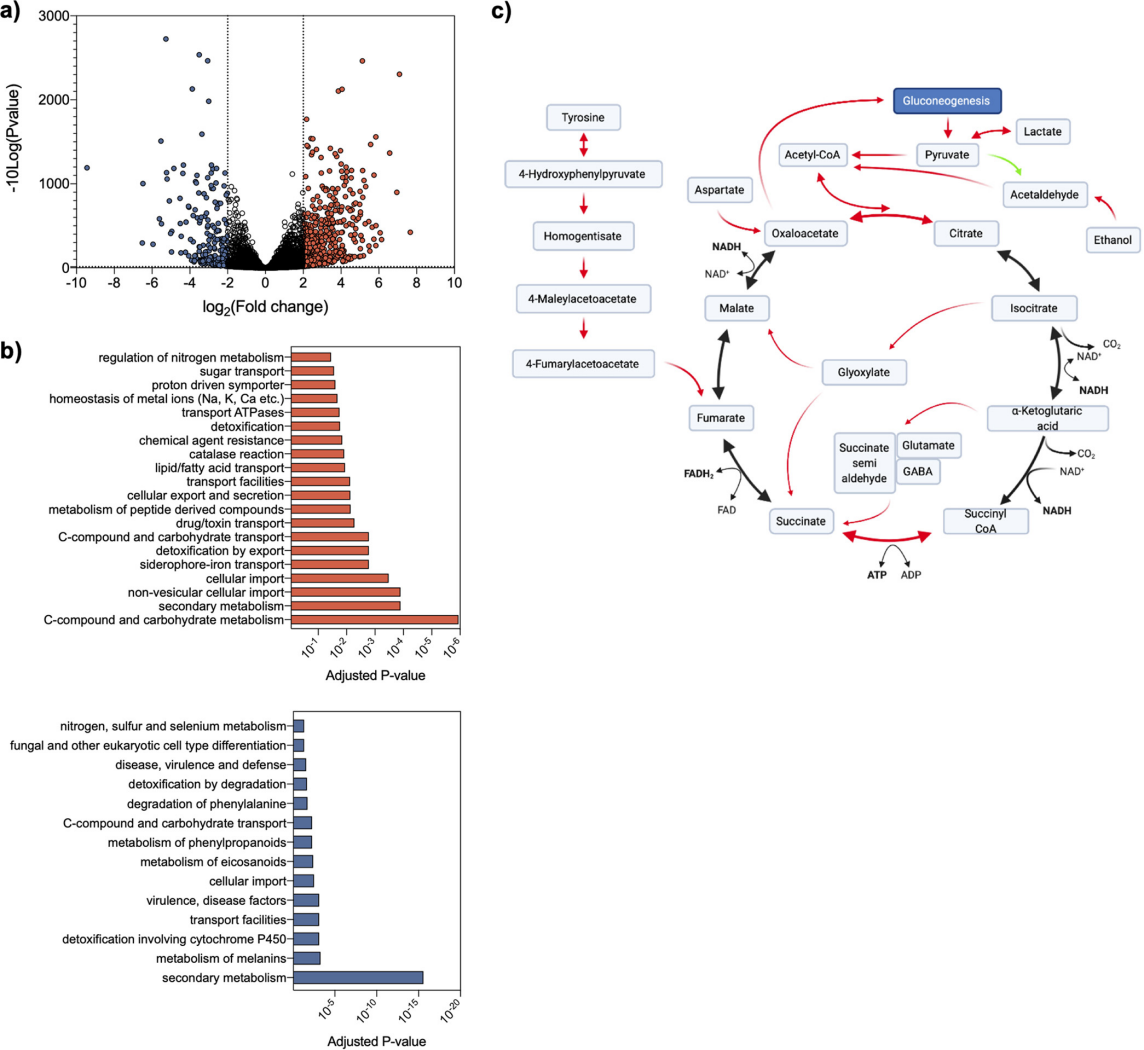
Transcriptome analysis of A. fumigatus Δ*ffmA* in the absence of Congo red exposure. (a) Volcano plot highlighting upregulated and downregulated genes (in red and blue, respectively). Cutoffs were set as a *P* value of <0.05 and a fold change difference of 2. (b) Enriched FunCat terms. Terms enriched in upregulated genes are in red, and those enriched in downregulated genes are in blue. (c) Overview of differentially regulated primary metabolism in the Δ*ffmA* mutant. Red and green arrows represent up- and downregulated processes, respectively. FAD, flavin adenine dinucleotide; FADH_2_, reduced flavin adenine dinucleotide.

Since the Δ*ffmA* mutant was the only selected mutant exhibiting reduced growth in the absence of CR, we assessed the FfmA regulon under control conditions before initiating an analysis of the genetic causes of the sensitivity to CR. FunCat analysis on the transcriptome sequencing (RNA-seq) data obtained for the *ffmA*-null mutant in the absence of CR revealed that FfmA is a negative regulator of a large and highly interconnected network of genes involved in primary metabolism (Fig. 3a and b). Specifically, this network is linked to enzymes that feed and participate in the tricarboxylic acid (TCA) cycle (Fig. 3c). Notably, both the glyoxylate and γ-aminobutyric acid (GABA) shunts are highly upregulated, as is acetaldehyde production. These data suggest that this transcription factor mutant favors fermentative metabolism, hence the name FfmA (favors fermentative metabolism). The activation of the glyoxylate and GABA shunts is likely to stimulate succinate biosynthesis, which in turn would be expected to result in increased reactive oxygen species (ROS) production via the conversion of succinate to fumarate by succinate dehydrogenase (34–36). Consistent with the cell experiencing oxidative stress upon the loss of FfmA, the genes encoding thioredoxin reductase (Trr1; AFUB_069890), the thioredoxin TxrA (AFUB_058890), a number of peroxidases (AFUB_036900, AFUB_062560, and AFUB_092440), and catalases (CatA; Cat2 and AFUB_017280) were significantly upregulated by more than 2-fold. Increased oxidative stress combined with the putative defect in carbon metabolism identified in the RNA-seq data could explain the growth defect observed in the Δ*ffmA* mutant (Fig. 3c and d). Interestingly, the genes associated with secondary metabolite biosynthesis that were upregulated in response to CR in the wild-type (WT) isolate differed markedly in the Δ*ffmA* mutant (https://doi.org/10.48420/14252768), suggesting that the fumagillin, pseurotin A, and fumisoquine clusters regulated by FfmA may be associated with oxidative stress.

### Transcriptome analysis indicates that the resistance to CR results from a combination of factors.

In a previous study, we were able to identify key factors that contribute to drug resistance using comparative transcriptomics (37). For this study, we sought to interrogate only those expression changes that were common in TF-null mutants with similar susceptibility phenotypes. In the sensitive isolates, 130 genes were commonly upregulated and 77 were commonly downregulated in response to CR, whereas in the resistant isolates, 249 were upregulated and 303 were downregulated (Fig. S5a and b). The functional categories “transmembrane transport” and “*N*-acetyltransferase activity” were enriched among downregulated genes in resistant isolates, while they were enriched among upregulated genes in the sensitive strains. In contrast, the functional category “extracellular region” was specific to resistant mutants and absent from the sensitive mutants. Interestingly, the gene expression of the resistant mutants was similar to that of the parental strain incubated in the presence of a high concentration of CR, suggesting that the drug concentration is the main driver of the modification of gene expression induced by the presence of the drug. For genes coregulated in the resistance or sensitivity to CR, significant GO terms include secondary metabolism (including siderophore biosynthesis), cell wall-related processes (organization and biosynthesis), membrane components, and cellular transport (Fig. S5c and d).

10.1128/mBio.00863-21.5 FIG S5 RNA-seq analysis of TF mutants. (a and b) Venn diagrams to identify genes commonly differentially regulated in sensitive (a) and resistant (b) isolates. (c and d) Gene ontology analysis by FungiFun2 and DAVID to identify functional categories enriched within commonly up- or downregulated genes in sensitive (c) and resistant (d) isolates. Download FIG S5, PDF file, 0.4 MB. Copyright © 2021 Liu et al. 2021 Liu et al. https://creativecommons.org/licenses/by/4.0/ This content is distributed under the terms of the Creative Commons Attribution 4.0 International license.

The resistance of the TF mutants to CR may be associated with an increased expression of specific transporters such as AFUB_017060, AFUB_057740, or, to a lesser extent, AFUB_101100, AFUB_022380, AFUB_016880, and AFUB_090450. In contrast, the downregulation of several transporters, such as AFUB_062170, AFUB_699705, AFUB_101120, AFUB_017190, AFUB_04880, or AFUB_081250, could be the reason for the increased sensitivity of the Δ*ffmA* and Δ*zipD* strains. However, some of these transporters, such as AFUB_057910, AFUB_006820, and AFUB_071390, which were highly downregulated in the Δ*ffmA* mutant, were not downregulated in the Δ*zipD* mutant, which suggests a different mode of susceptibility (Fig. S6). The functions of these transporters are not annotated, and a systematic gene deletion analysis will be required to confirm their putative function as drug efflux pumps.

10.1128/mBio.00863-21.6 FIG S6 Differentially regulated A. fumigatus transporters upon Congo red exposure. Shown is a heat map of predicted major facilitator superfamily (MFS) and ABC transporters (greater than 2- or less than −2-fold differentially regulated under at least one condition) in the A. fumigatus Δ*ffmA*, Δ*ace1*, *ΔzipD*, and Δ*crrA* mutants. Download FIG S6, PDF file, 0.1 MB. Copyright © 2021 Liu et al. 2021 Liu et al. https://creativecommons.org/licenses/by/4.0/ This content is distributed under the terms of the Creative Commons Attribution 4.0 International license.

The biosynthesis of secondary metabolites was influenced by the addition of CR, but these changes did not seem to have any relationship with their sensitivity or resistance to the drugs (https://doi.org/10.48420/14252768). For example, the pyripyropene A cluster, which was upregulated in the parental strain, is downregulated in both the sensitive Δ*ffmA* and resistant Δ*sltA* mutants irrespectively of their resistance to the drug. Similarly, the upregulation of genes involved in iron metabolism was not significantly different in the sensitive and resistant mutants and their parental strains (Fig. S3a). Accordingly, the addition of 0.2 mM the iron chelator bathophenanthrolinedisulfonic acid (BPS) or 5 mM FeSO_4_ did not modify the sensitivity of all strains to CR (Fig. S3b). The upregulation of plant-degrading glycosyl hydrolases such as cellulase, xylanases, and pectinases was noticed in all mutants (Fig. S4a). This upregulation was similar to that of the parental strain and only indicated a stress response consecutive to any type of growth inhibition and fungal starvation. As for plant glycosyl hydrolases, the enzymes of the ergosterol biosynthesis or the secondary metabolite clusters were not differentially expressed in the function of their resistance to CR (https://doi.org/10.48420/14252768). A similar conclusion was obtained with the expression of cell wall genes since the resistance or susceptibility of the 4 mutants studied was not associated with a clear-cut difference in the expression of these cell wall genes (Fig. S4b). This was true for enzymes for the synthesis of the sugar nucleotides or synthases as well as transglycosidases and glycosyl hydrolases. However, the level of phosphorylation of MpkA, which is a key player in the cell wall integrity pathway of A. fumigatus, of the TF mutants is affected by the presence of CR (Fig. S7). Both resistant mutants Δ*crrA* and Δ*sltA* had a higher MpkA phosphorylation level than the parental strain and the sensitive strains. This result showed that resistance to CR, like other cell wall stresses (38), was associated with increased phosphorylation of MpkA.

10.1128/mBio.00863-21.7 FIG S7 Immunoblot analysis of MpkA phosphorylation of the wild type and mutants in medium with CR. Anti-phospho-p44/42 MAPK or anti-p44/42 MAPK antibodies directed against phosphorylated MpkA and total MpkA were used to detect the phosphorylation of MpkA and total MpkA, respectively. Signal intensities were quantified using ImageJ software by dividing the intensity of MpkA-P by that of MpkA. Download FIG S7, PDF file, 0.6 MB. Copyright © 2021 Liu et al. 2021 Liu et al. https://creativecommons.org/licenses/by/4.0/ This content is distributed under the terms of the Creative Commons Attribution 4.0 International license.

As Congo red is known to have high oxidative potential, the hypersensitivity observed in the Δ*ffmA* mutant may be, in part, a consequence of combined oxidative stresses. Indeed, the expression of many oxidoreductases was highly stimulated in the presence of CR, and overexpression of many anti-ROS enzymes and molecules was seen in the resistant mutants (Fig. S8a). For example, in the resistant strains, we noticed the upregulation of oxidoreductases such as the superoxide dismutase SOD1 (AFUB_056780) and a catalase (AFUB_017280) and thioredoxin and glutaredoxin systems such as glutaredoxin (AFUB_006480) and thioredoxin peroxidase (AFUB_062560). However, some of the proteins acting on ROS were also downregulated. Even though it was impossible to delineate the ROS pathways associated with the impact of CR on A. fumigatus, it was clear that there was an interaction or at least a link between the presence of intracellular ROS and the toxicity of CR. In yeast, *sod1* mutants were highly susceptible to CR (39). In summary, specific gene expression was associated with the presence of CR in the fungal environment. The analysis of the mutant transcriptome data demonstrated that no single gene expression pathway was directly linked to differential susceptibility to Congo red, which suggested that resistance to CR results from a combination of factors.

10.1128/mBio.00863-21.8 FIG S8 ROS and polarization genes. The expression of genes involved in resistance to reactive oxidant stress (a) and genes associated with the polarization of filamentous growth (b) was determined. Download FIG S8, PDF file, 0.10 MB. Copyright © 2021 Liu et al. 2021 Liu et al. https://creativecommons.org/licenses/by/4.0/ This content is distributed under the terms of the Creative Commons Attribution 4.0 International license.

### Susceptibility of mutants to CR does not correlate with susceptibility to other drugs acting on the cell wall.

To assess if transcription factors regulating resistance to CR are also linked to susceptibility to other cell wall inhibitors such as caspofungin (caspo), the chitin synthase inhibitor nikkomycin Z (nikko), and calcofluor white (CFW), all four previously uncharacterized TF-null mutants and the Δ*rlmA* and Δ*crzA* isolates were assessed for their ability to grow in the presence of these agents. Consistent with our hypothesis that FfmA has a general role in regulating central metabolism leading to a reduction in the production of all cell wall constituents, the Δ*ffmA* isolate is hypersensitive to all cell wall inhibitors. The sensitivity profiles of the other mutants uncovered striking differences between CR and the other agents acting on the cell wall and highlighted the different roles played by each TF in the control of cell wall construction in A. fumigatus. The CR-resistant Δ*crrA*, Δ*sltA*, and Δ*crzA* mutants were less susceptible to the chitin binding agent CFW and the chitin biosynthesis inhibitor nikkomycin than the parental strain (Table 2). The phenotypic profiles of the Δ*zipD* and Δ*rlmA* mutants were similar to each other but opposite those of the Δ*crzA* and Δ*crrA* mutants. The fact that the Δ*crzA* and Δ*crrA* isolates phenocopied each other in this screen suggested that there may be a significant overlap in the regulons of the TFs disrupted in these strains. Interestingly, unlike the Δ*crzA* and Δ*crrA* isolates, the Δ*sltA* isolate was hypersensitive to caspofungin, suggesting that SltA is also responsible for regulating glucan synthesis or another factor governing adaptation to caspofungin exposure. This is evident in the transcriptomic data as genes critical for the synthesis of alpha-glucan are more highly expressed in the Δ*sltA* mutant than in any other isolate. These data corroborated the transcriptome results and confirmed the pleiotropic antifungal role of Congo red, which is not exclusively associated with cell wall inhibition.

**TABLE 2:** Susceptibility of the parental strain and TF mutants mostly affected by CR incubation to cell wall antifungal drugs

Strain	Gene ID	Description	Sensitivity^*a*^			
			CR	CFW	Caspofungin	Nikkomycin Z
1B4	AFUA_1G06900	C_2_H_2_ transcription factor (CrzA)	+ +	+ + + +	+/−	+ + +
1D4	AFUA_1G15230	C6 transcription factor (CrrA)	+ + + +	+ + +	+/−	+ + +
1F4	AFUA_2G03280	bZIP transcription factor (ZipD)	− − − −	− − − −	+ + +	− − −
1G9	AFUA_2G10550	C_2_H_2_ transcription factor (FfmA)	− − − −	− − − −	− − − −	− − − −
2C5	AFUA_3G08520	SRF-type transcription factor (RlmA)	− − −	− − −	+ + +	+/−
2C7	AFUA_3G08010	C_2_H_2_ transcription factor (Ace1)	+ + +	+ + +	− − − −	+ + +

a +, more resistant than the parental strain; −, more sensitive than the parental strain; +/−, no different from the parental strain.

### The amount of surface galactosaminogalactan increases in mutants resistant to CR.

Because of the suggested role of CR as a cell wall inhibitor and the differential sensitivities to cell wall inhibitors of the TF mutants, the composition of the cell wall of the parental strain and mutants grown in the presence or absence of CR was later investigated (Table 3). In the absence of CR, the compositions of the cell wall of the resistant and sensitive mutants were different. CR-resistant mutants had a lower concentration of chitin and a higher concentration of glucans than sensitive mutants. When the parental strain and the mutants were grown in the presence of CR, the amounts of glucose, galactose, and mannose were significantly reduced in all strains. In contrast, the percentage of GlcNAc increased in the presence of CR. These results indicated that the cell wall glucans and galactomannan were highly reduced in the presence of CR, while the amount of chitin was significantly increased, a classical response to many stresses in filamentous fungi (40, 41). However, the same trend was observed for all mutants without any significant correlation with sensitivity or resistance to CR. These results indicated that CR modified the synthesis of cell wall glucans, chitin, and galactomannan, but these cell wall modifications were not directly correlated with an induction of resistance or sensitivity to CR in our TF mutants.

**TABLE 3:** Monosaccharide composition of the cell wall of the parental strain and mutants grown in liquid culture in medium without CR or with CR^*a*^

Monosaccharide	Mean % monosaccharide composition ± SD									
	Δ*ku80*		Δ*zipD*		Δ*ffmA*		Δ*crrA*		Δ*ace1*	
	−	+	−	+	−	+	−	+	−	+
Mannose	5.8 ± 0.7	2.1 ± 0.1*	5.4 ± 1.2	1.9 ± 0.1*	5.1 ± 1.5	3.1 ± 1.0*	6.3 ± 0.4	2.6 ± 0.7*	5.5 ± 1.1	3.0 ± 0.6*
Glucose	55.8 ± 0.9	50.0 ± 5.0	52.1 ± 2.4	51.8 ± 5.7	54.7 ± 7.8	50.3 ± 5.7	62.2 ± 7.0	48.6 ± 5.4*	63.6 ± 6.7	52.8 ± 2.3*
Galactose	7.1 ± 2.0	2.1 ± 0.3*	7.1 ± 3.0	1.4 ± 0.2*	5.2 ± 1.3	2.0 ± 0.6*	8.5 ± 0.3	3.1 ± 1.1*	8.3 ± 1.4	4.2 ± 1.0*
GalNAc	6.9 ± 2.0	7.4 ± 1.0	6.3 ± 2.3	3.9 ± 0.4*	6.1 ± 4.6	4.4 ± 0.6*	4.6 ± 0.8	8.8 ± 1.3*	2.5 ± 1.1	9.7 ± 1.5*
GlcNAc	24.4 ± 1.5	38.4 ± 4.9*	29.1 ± 3.7	40.9 ± 5.5*	29.0 ± 6.0	40.2 ± 6.7*	18.5 ± 5.5	36.7 ± 5.8*	20.1 ± 3.5	30.4 ± 2.3*

a The values represent the means ± SD (*n* = 9). Values of monosaccharide amounts in the cell wall of mycelium grown in liquid culture in the presence of CR (+) versus the values obtained in the absence of CR (−) marked with * are significantly different (*t* test).

**FIG 4:**
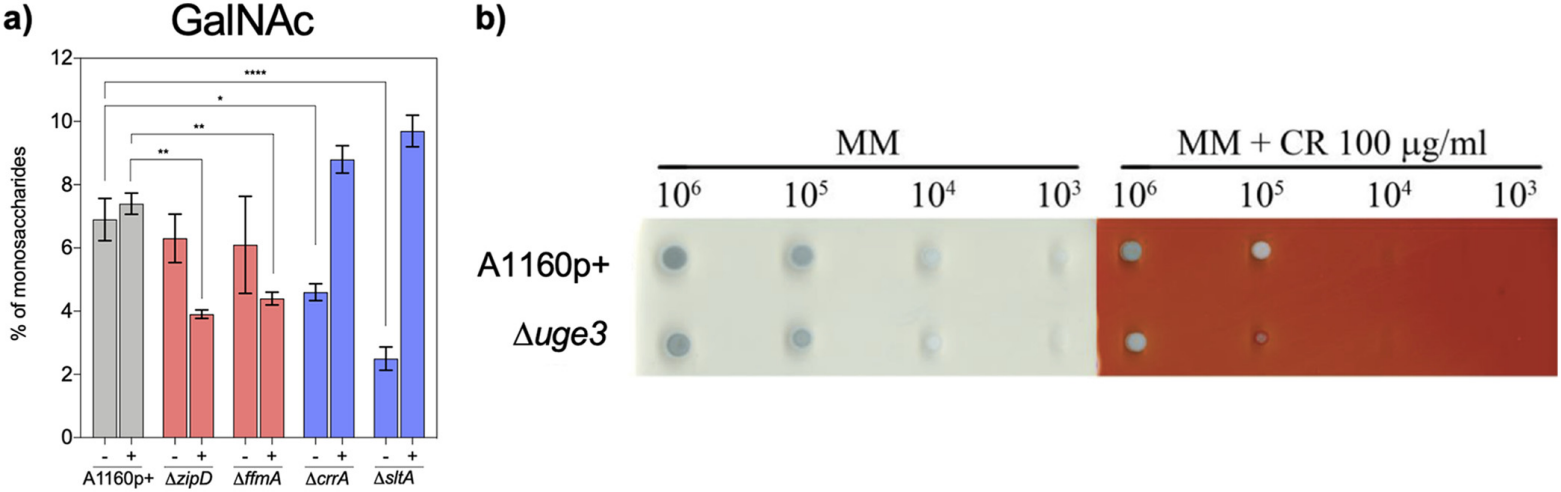
Influence of galactosaminogalactan (GAG) on resistance to CR. (a) The cell walls of the CR-resistant mutants are richer in galactosamine (which is an indication of the GAG content) than those of the parental strain and the CR-sensitive Δ*crrA* and Δ*sltA* mutants. The concentration of galactosamine plus *N*-acetylgalactosamine is estimated as a percentage of the galactosamine of the total extracted cell wall components. Statistical significance was assessed by two-way analysis of variance (ANOVA) (*, *P* < 0.05; **, *P* < 0.01; ***, *P* < 0.001). (b) A Δ*uge3* mutant deficient in GAG is more susceptible to CR (100 μg/ml) than the parental strain.

In contrast, the GalNAc content (indicative of the presence of cell wall GAG) of the sensitive Δ*zipD* and Δ*ffmA* mutants was reduced in the presence of CR, while the percentage of GalNAc in the cell wall of the resistant Δ*crrA* and Δ*sltA* mutants increased to 7.5% and 8.1% of the total carbohydrates, respectively (Fig. 4a). Particularly due to its sticky property, the larger amount of GAG in the resistant mutant could be responsible for the higher binding capacity of CR to these resistant strains. Accordingly, the Δ*uge3* mutant, which does not produce GAG, was more sensitive to CR (Fig. 4b). The direct link between sensitivity to CR and modification of the surface GAG suggested that the antifungal activity of CR could be associated with modulation of cell wall permeability.

### Permeability of the cell wall to liposomes is reduced in CR-resistant mutants.

**FIG 5:**
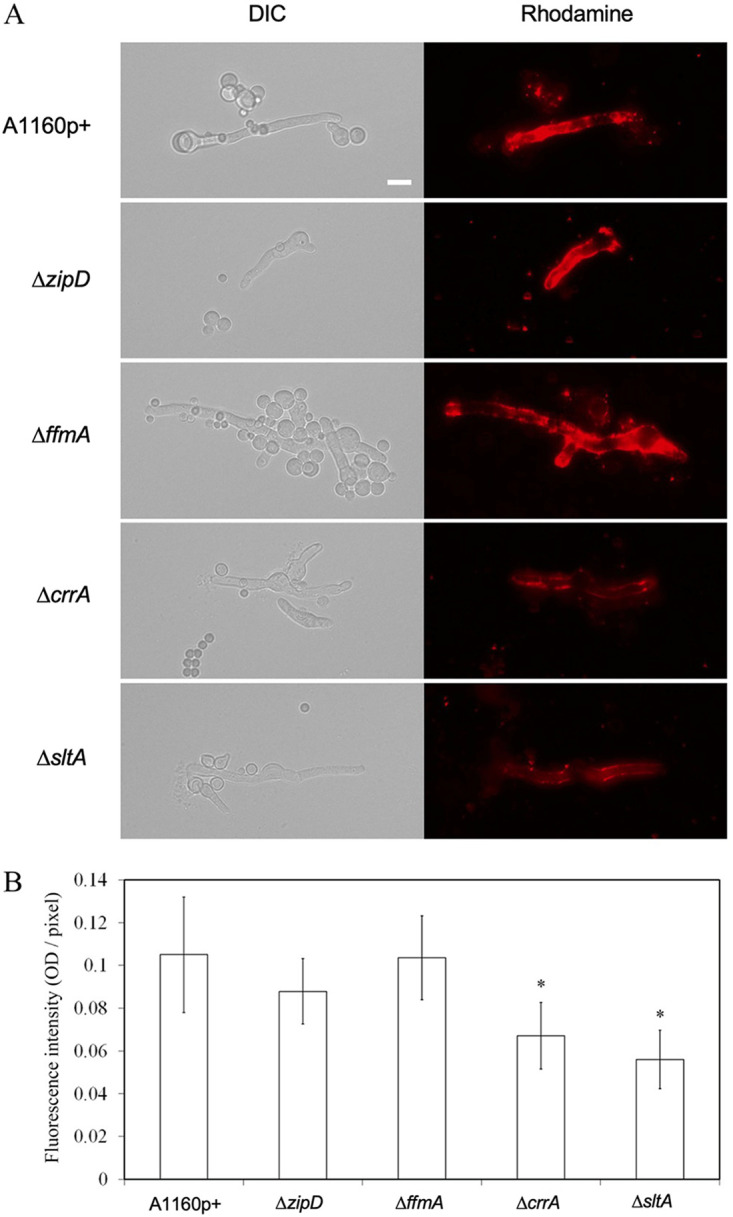
Cell wall permeability of CR mutants. (A) DIC and fluorescence images of mycelium treated with liposomes labeled with 1% rhodamine were captured by fluorescence microscopy. Bar, 10 mm. (B) The fluorescence intensity was quantified by ImageJ 1.8.0 and based on 9 randomly selected images. The columns marked with * are significantly different. OD, optical density.

One major factor that could contribute to CR sensitivity is its ability to cross the cell wall into the cell. To investigate the permeability of the cell, we used liposomes labeled with rhodamine. Liposomes could penetrate the cell wall of the wild type and different mutants after 3 h of incubation at 37°C. The fluorescence intensity of rhodamine, which is an indication of the binding of the drug to the cell wall, was lower in the CR-resistant Δ*crrA* and Δ*sltA* mutants than in the parental strain (36 and 47% lower, respectively [*P* < 0.01]) (Fig. 5A and B). There was no difference between the parental isolate and CR-sensitive mutants. The results also indicated that the binding of the liposomes to the cell wall and/or their intracellular penetration was affected by the deletion of the TF. The increased permeability of the parental and sensitive mutant strains to liposomes was in agreement with an increased sensitivity of these strains to CR. In contrast, the lower influx of liposomes in the resistant mutants fits well with an increased binding of the drug to the cell wall.

### Conidial germination asynchrony is a novel mechanism that facilitates drug tolerance in A. fumigatus.

**FIG 6:**
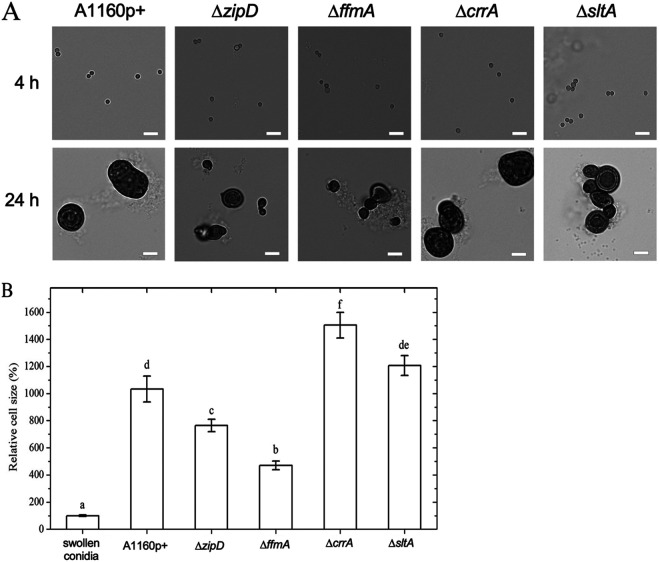
Swollen conidia of the parental strain and TF mutants in liquid medium with CR without shaking at 4 h and 24 h. (A) Cell sizes of swollen conidia and Quasimodo cells. The cell morphology of every strain was observed at different time points. Bars, 10 mm. The sizes of the cells were analyzed by ImageJ 1.8.0. (B) Quantification of Quasimodo cell size. Values represent the means ± SD (*n* > 15 and from at least 3 independent replicates). The size of swollen conidia was set as 100%. Values marked with different letters are significantly different from each other (*P* < 0.05).

The germination of A. fumigatus conidia is a dimorphic event. Initially, conidia undergo isodiametral swelling until they reach 5 μm, at which point a polarized germ tube is formed. When the conidia of the wild type were inoculated in buffered MM with CR (100 μg/ml) and incubated at 37°C without shaking, the conidia swelled normally for up to 4 h of incubation (Fig. 6A). However, after this initial swelling phase, germ tubes did not form. Instead, the conidia continued to swell, becoming 7.4 to 14.5 times larger than those seen in medium without CR (Fig. 6B). Moreover, the CR-treated swollen conidia had a modified cell wall, which was 3.4 to 5.2 times thicker than that in the absence of CR. We called these abnormal cells Quasimodo cells. Accordingly, the RNA-seq data showed that genes important for polarization were downregulated in the parental isolate and further downregulated in the resistant isolates (Fig. S8b), while the mTor pathway was not affected (https://doi.org/10.48420/14252756).

After 24 h of incubation with CR, Quasimodo cells reached their maximal size. Their size did not increase after 24 h. At 2 h, the Quasimodo cells of the sensitive Δ*zipD* and Δ*ffmA* mutants were smaller than those of the wild type and reached averages of 0.75 times (*P* < 0.05) and 0.5 times (*P* < 0.01) the size of the parental strain incubated under the same conditions, respectively. In contrast, the Quasimodo cells of the resistant Δ*crrA* and Δ*sltA* mutants were larger than the Quasimodo cells of the parental strain and sensitive TF mutants. In the presence of 100 μg/ml of CR, the Quasimodo cells of the Δ*crrA* and Δ*sltA* mutants were 1.5 times (*P* < 0.01) and 1.2 times (*P* < 0.05) larger than those of the parental strain, respectively (Fig. 6B). All Quasimodo cells were dead since they were not able to germinate when transferred to fresh culture medium (data not shown).

**FIG 7:**
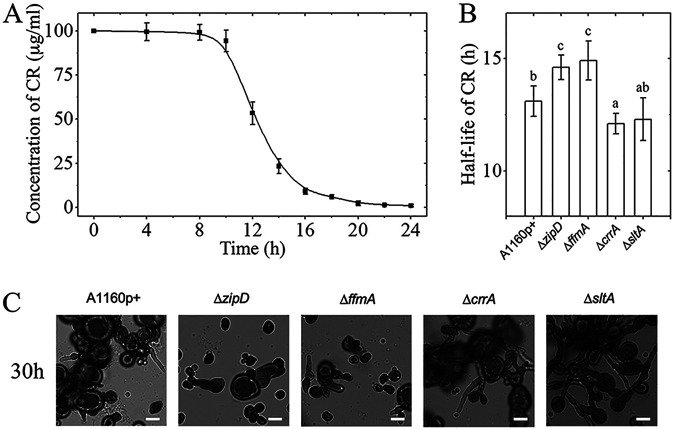
Characteristics of the mycelial growth of the TF mutants and the parental strain in the presence of CR (100 μg/ml). (A) Kinetics of the concentration of CR in medium as an indicator of the uptake of CR by germinating conidia of the parental strain. (B) Time (hours) after the beginning of germination when the CR concentration reaches half of the original concentration of CR in the medium of the mutants and the parental strain. The values represent the means ± SD (*n* = 9). (C) Morphology of every mutant observed after 30 h of growth in medium with CR (100 μg/ml) at 37°C. Bars, 10 μm.

For all strains, CR adhered to the cell wall of the germinating conidia. Moreover, CR was observed intracellularly in Quasimodo cells of all strains (Fig. 7A). We then assessed the amount of CR remaining in the medium after the growth of the different mutants (the opposite of the amount of drug absorbed by the fungus). The decrease of the initial CR concentration of 100 μg/ml was measured in culture supernatants at regular time intervals up to 24 h. For the parental isolate, the concentration of CR in the culture supernatants started to decrease after 8 h of incubation at a time corresponding to the swelling of the conidium and the initiation of the Quasimodo phenotype (Fig. 7A). Half of the initial CR concentration in the medium of the parental strain was reached after 13.1 h (Fig. 7B). The CR concentration in the culture supernatants of the sensitive Δ*zipD* and Δ*ffmA* mutants decreased more slowly than in the wild type, and the half-concentration of CR in these sensitive mutants was seen after 14.6 h and 14.9 h, respectively. In contrast, the Quasimodo cells of the resistant Δ*crrA* and Δ*sltA* mutants removed CR more rapidly from the medium, with a 50% reduction in CR levels being reached after 12.3 h and 12.1 h, respectively (Fig. 7B). Interestingly, we observed that not all conidia formed Quasimodo cells. In the presence of 100 μg/ml, 10% of conidia of the parental strain and the Δ*ffmA* mutant and 5% of the conidia of the Δ*zipD* mutant remained with a normal morphology of resting conidia. However, these conidia were not able to germinate subsequently. In contrast, 11% of the conidia of both resistant mutants Δ*crrA* and Δ*sltA*, which remained with a morphology of resting conidia after CR treatment, were all able to germinate afterward (Fig. 7C). These data suggest that Quasimodo cells act as chemical detoxifiers to prepare a drug-free environment for late-germinating conidia. One mechanism by which mutants were able to better adapt to the presence of CR was associated with their ability to more rapidly detoxify the environment.

### A novel growth effect by priming A. fumigatus with CR.

**FIG 8:**
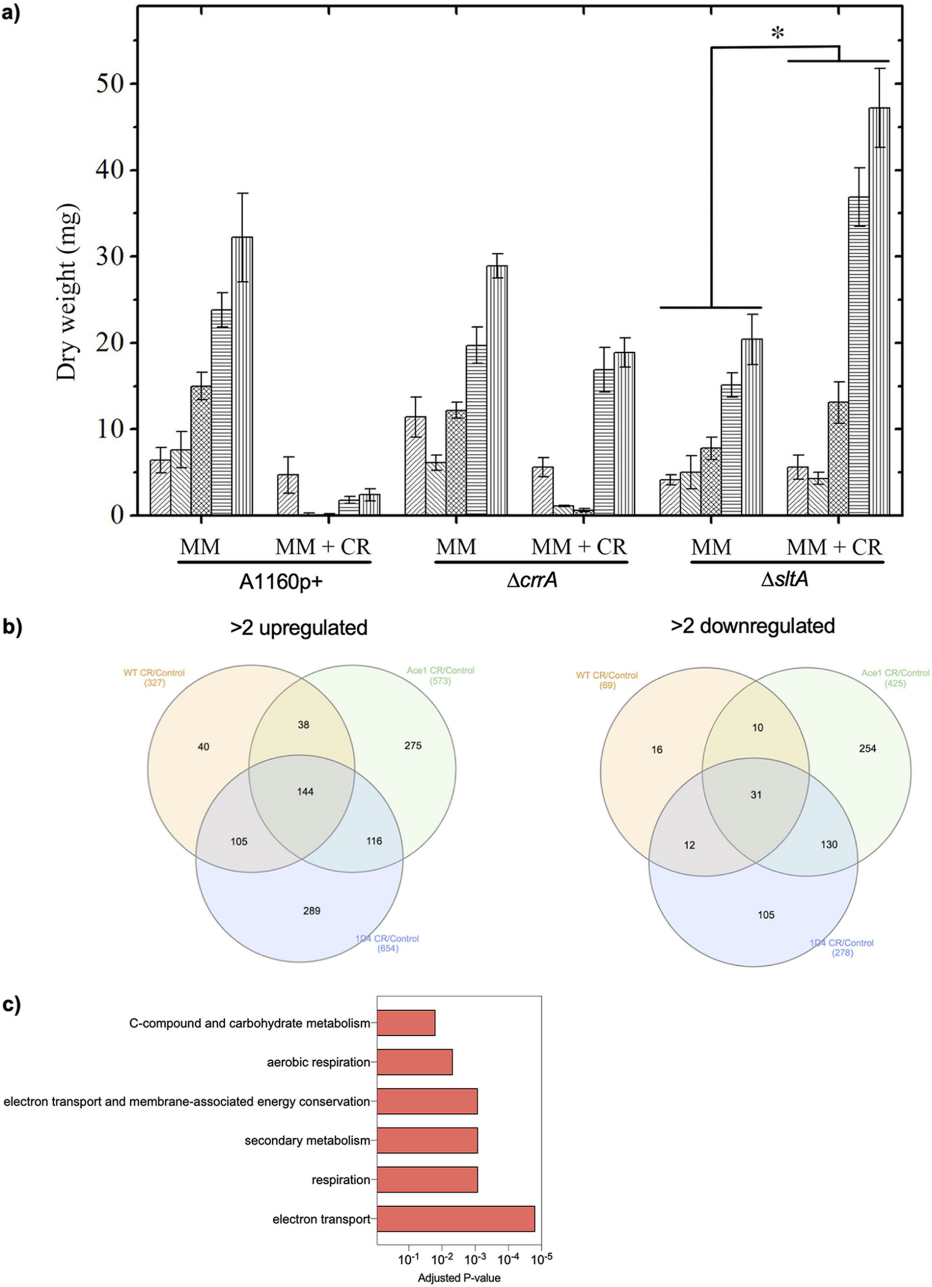
Stimulation of growth of the Δ*sltA* resistant strain by CR. (a) Growth of the wild type and resistant Δ*crrA* and Δ*sltA* mutants in liquid buffered MM medium in the absence or presence of CR at 300 μg/ml under shaken conditions after priming the mutants with CR. The values represent the means ± SD (*n* = 9) of growth for 0, 24, 48, 72, and 96 h. Values marked with an asterisk are significantly different (*P* < 0.05). (b) Venn diagram showing the different populations of genes up- and downregulated in the mutants and the parental strain. (c) KEGG enrichment analysis of upregulated genes for 4 days of growth in the Δ*sltA* mutant.

The resistant Δ*crrA* and Δ*sltA* mutants were assessed for growth over a time course of 5 days in the absence or presence of 300 μg/ml CR in liquid medium with shaking. In the presence of 300 μg/ml CR, the growth of the parental strain was completely inhibited up to 120 h (Fig. 8a). Under the same conditions, the growth of the Δ*crrA* mutant mirrored that of the wild type up to 72 h. However, after this time, the biomass of the mutant increased dramatically, almost reaching the levels of the untreated cultures. In contrast, the growth of the Δ*sltA* mutant appeared unaffected up to 48 h. Intriguingly, after this time, the growth of the Δ*sltA* mutant was strongly enhanced and reached almost 2.5 times the growth in the absence of the drug (Fig. 8a). The observations from both strains are consistent with our observations that after 48 h, CR is absorbed by Quasimodo cells. The stimulation of the growth of the Δ*sltA* mutant cannot be simply explained by the metabolization of the CR itself, since milligrams of dry weight gains were seen, while only microgram quantities of CR were added to the medium. These results indicate that the presence of CR in the medium stimulated the metabolism of the resistant Δ*sltA* mutant, enhancing fungal growth. Here, we have named this response drug-induced growth stimulation (DIGS).

The expression of genes associated with DIGS was investigated by RNA-seq using mycelia grown for 4 days in the presence or absence of CR. These transcriptomic data suggested that protein synthesis was stimulated in the Δ*sltA* mutant grown in the presence of the drug (Fig. 8b and https://doi.org/10.48420/14252759). The expression of genes involved in ribosome biogenesis (GO:0042254), cellular amino acid metabolic processes (GO:0006520), translation (GO:0006412), and protein folding (GO:0006457) was only upregulated only in the resistant Δ*sltA* mutant in the presence of CR at 4 days (Fig. 8c). DIGS of the parental strain and the sensitive Δ*ffmA* and Δ*zipD* mutants was investigated in the presence of 50 μg/ml CR. Under these conditions, no DIGS of these strains was noticed after a week of culture (data not shown). After CR stress, only the Δ*sltA* mutant showed DIGS, which resulted from enhanced amino acid synthesis, ribosome biogenesis, and protein synthesis.

## DISCUSSION

CR was previously associated with β-1,3-glucans, disorganizing the fungal cell wall structure. The fungus responds to CR by modifying its cell wall composition. Changes in the glucan and chitin contents of the cell wall consecutively to the deletion of glucan and chitin synthases have been reported to lead to hypersensitivity to CR (13). Most of the recent biochemical investigations on the antifungal activity of Congo red come from studies on CRH yeast mutants, which are hypersensitive to Congo red (39, 42, 43). Previous studies suggested that the Crh enzymes generate cross-links between the reducing ends of the chitin chains and the nonreducing ends of the β-1,3-glucan and β-1,6-glucan chains because there is no chitin covalently bound to β-1,3-glucan in yeast CRH1/2-null mutants. A catalytic mechanism has been proposed, which suggested that the transglycosylase activity of these proteins would involve the cleavage of β-1,4-glycosidic linkages of the chitin and the subsequent attachment of the fragment from the donor molecule with the newly formed reducing end onto the O-4 hydroxyl group of the acceptor molecule by a β-1,4-glycosidic bond. However, the formation of these linkages could not be shown *in vitro* with recombinant proteins and different types of acceptors. The CRH genes of the GH16 family have been found in many other fungal genomes, including *Aspergilli*. However, in contrast to S. cerevisiae, systematic analyses of CRH mutants of A. fumigatus did not show increased susceptibility to CR and did not show any growth phenotypes (17, 18). These results suggest that the function of CRH proteins may have been overemphasized in fungi and overlooked in cell wall construction. Moreover, increased expression of the *Crh1* (AFUB_015530) and *Crh5* (AFUB_095070) genes in A. fumigatus was observed upon CR exposure, but this was seen irrespective of the mutants’ susceptibility to Congo red. In S. cerevisiae, it was suggested that Crh enzymes could act not only as heterotransglycosylases attaching chitin chains to β-glucans but also as homotransglycosylases forming chitin-to-chitin cross-links. The increase in the chitin content of the cell wall of A. fumigatus submitted to CR is in agreement with such a putative biochemical function.

In our study, four transcription factor mutants displayed high levels of resistance to CR, namely, Δ*crzA*, Δ*crrA*, Δ*crrB*, and Δ*sltA*. The CrzA TF has already been studied, and the Δ*crzA* mutant has been shown to be CR resistant (26). The SltA TF has been studied in Aspergillus nidulans. SltA is highly conserved in the Ascomycota phylum, with no identifiable homolog in S. cerevisiae and other yeastlike fungi (44). The null allele of *sltA* causes sensitivity to elevated concentrations of monovalent cations such as sodium, lithium, rubidium, and potassium, among others, and to divalent cations such as manganese and magnesium. Another important phenotype is the sensitivity of the *sltA*-null mutant to alkalinity. Its susceptibility to CR and other cell wall-associated drugs has not been previously investigated in molds. However, recently, it was shown that the *sltA*-null strain is hypersensitive to the azoles (45). The fact that an *sltA*-null mutant of Colletotrichum gloeosporioides inhibited appressorium formation suggests that SltA may be involved in cell wall synthesis (46). Two other TFs have not been studied to date and were named CrrA and CrrB. The *crrB* gene was annotated as a putative ortholog of elongin A, a member of the ubiquitin ligase complex involved in DNA repair, but the homologies of the sequence are too low (3e^−08^) to consider them orthologous genes. The *crrA* gene is homologous to the *vad5* gene of Neurospora crassa, which is associated with hyphal growth and conidium formation in N. crassa (47). However, the susceptibility of the *vad5* mutant to antifungals has not been investigated.

The mutants most susceptible to CR were Δ*zipD*, Δ*ffmA*, and Δ*rlmA*. The Δ*rlmA* mutant was previously shown to be CR sensitive (23). The Δ*zipD* mutant was shown to be associated with calcium metabolism but with a sensitivity to CR opposite that of the Δ*crzA* mutant (24). The most sensitive TF mutant, Δ*ffmA*, was the only mutant showing a strong growth reduction in the absence of CR. Our RNA-seq analysis suggested that this TF is a key growth regulator in A. fumigatus and favors fermentation metabolism. Three other TFs (2B3, 2G7, and 5H9), which were strong orthologs of the gene *sppA* in Aspergillus oryzae and the genes *steA* and *mtfA* in A. nidulans and A. fumigatus, were associated with sexual reproduction and secondary metabolite production (48–51). Finally, two global regulators of general metabolism dealing with pH adaptation (PacC [2B5]) and carbon catabolite repression (CreA [1G11]) have also been shown to be involved in cell wall construction and are logically associated with CR susceptibility (52, 53). Our study has led to the discovery of three important new paradigms in antifungal drug resistance not associated with mechanisms classically implicated in drug resistance.

First, we have shown that the heterogeneity of the conidial population promotes the survival of A. fumigatus in the presence of deadly chemicals in a manner consistent with antibiotic persistence (54). Regrowth of the persister cells is facilitated by the detoxification of the environment by a subpopulation of conidia that sacrifice themselves by absorbing and clearing the drug from the medium. The heterogeneity of Aspergillus nuclei has been investigated in Aspergillus niger (55). Although the heterogeneity of an Aspergillus population has been mainly studied in A. niger and A. oryzae due to their biotechnological purposes, cellular heterogeneity has been seen among A. fumigatus conidium populations (56). Significant differences in the time to germination have been noticed, while all these uninucleate conidia originate from the same conidiophore (57). The concept of nuclear autonomy for multinucleate organisms is considered to have an essential role during infection or resistance to drug treatments (58, 59). The occurrence of Quasimodo cells has highlighted the role of conidial heterogeneity in tolerance to drugs. However, such cells have not been found after *in vitro* treatment of A. fumigatus with other cell wall-targeted antifungal drugs such as calcofluor white or echinocandins. The mechanism of self-sacrificial detoxification and persistence clearly has the potential to mediate tolerance to other drugs; however, to our knowledge, this is yet to be described in filamentous fungi.

Second, the permeability of the fungus to the drug and the associated binding of the drug to the cell wall are essential for drug efficacy. The role of cell wall permeability and the influx of amphotericin B (AmBisome) in Candida albicans was recently shown (60). Assessment of cell wall permeability with rhodamine-loaded liposomes is of increased interest for CR since this drug, like liposomes, can form amphipathic structures. Our experiments with liposomes confirmed that cell wall permeability plays a major role in CR intake. However, our transcriptome data do not show differential regulation of the plasma membrane H^+^-ATPase PMA1 in response to CR, nor in any of the mutants. In contrast, GAG was shown to be a major player in cell wall permeability since GAG-deficient mutants are more susceptible to CR, while resistant mutants have a larger amount of GAG in their cell wall. GAG, which is composed of α1-4-linked galactose and α1-4-linked GalNAc and GalNH_2_ residues, is a surface polysaccharide in the cell wall of A. fumigatus with strong binding properties to a variety of molecules and cells (61–64). CR stress is also linked to an increase in chitin synthesis. Deletion of cell wall biosynthesis genes or antifungal treatment results in increased chitin levels. However, the role of increased chitin in the permeability of the cell wall has not been investigated yet. Interestingly, the Δ*sltA* mutant is also hypersensitive to itraconazole and amphotericin B (22) (data not shown). This may indicate that the Δ*sltA* mutant has additional defects in its membrane architecture and/or permeability that lead to enhanced susceptibility to sterol-associated antifungal drugs. Similarly, both sensitive mutants Δ*ffmA* and Δ*zipD* were also hypersensitive to itraconazole (22). Even though the role of GAG has been demonstrated, an additional effect of a direct alteration of the efflux pumps on drug permeability cannot be discarded. Our data suggest that the binding and crossing of the cell wall and cell membrane by antifungal molecules are multifactorial phenomena that may not be directly connected to the mode of action of the antifungal, which remains to be investigated. While we cannot certainly identify the primary site of action of CR, we can speculate that the inhibition of cell wall polysaccharide by CR is not due to a single direct effect but is a result of a combination of downstream effects.

Third, we identified a new growth phenomenon in response to priming with an antifungal drug, called DIGS. This priming may result in extensive growth stimulation in response to prior stress (including antifungals). In humans during trained immunity and in yeast, the activation of specific metabolic pathways that result in the epigenetic rewiring of cellular functional programs has been reported to control the memory of environmental stresses (65–67). Recent data with other fungal species suggest that this environmental memory is not limited to a very specific strain and antifungal but seems a general phenomenon in fungi (68–70). In C. albicans, memory has been demonstrated by cross-protection under stressed conditions (71). In the case of A. fumigatus, DIGS has previously been found in response to itraconazole and rotenone (34). This paradigm should be investigated in more detail since the apparent lack of drug specificity may have fundamental implications for antifungal therapy.

This study on the resistance of A. fumigatus to Congo red has shown that the mechanisms controlling antifungal drug resistance are more complex than the establishment of single genetic mutations of the target genes or the overexpression of efflux pumps (72). The mechanism responsible for this metabolic change remains to be identified. We currently have a limited understanding of whether similar responses occur *in vivo* during antifungal treatment. We require a better understanding of fungal responses to drugs *in vivo* to understand drug tolerance or the failure of drug treatment in patients. Moreover, it would be essential to know if DIGS is sustainable without *de novo* drug training. This especially indicates that the introduction of an antifungal drug during fungal growth induces a complete rewiring of global metabolism in addition to the inhibition of the drug target.

## MATERIALS AND METHODS

### Fungal strains.

The TF knockout library was generated in the strain MFIG001 (27, 73), which is deficient in nonhomologous end joining. Briefly, gene knockout cassettes were generated using a fusion PCR approach and used to transform protoplasts as described previously (22, 74). The successful integration of single homologous integration was validated by PCR. The Δ*uge3* mutant was a kind gift of F. Gravelat and D. Sheppard.

### Complementation of gene knockout isolates.

Knock-in (KI) cassettes were designed to complement the deleted genes for mutants 1D4 (*ccrA* [AFUB_014780]), 2C7 (*sltA*/*ace1* [AFUB_041100]), and 1F4 (*zipD* [AFUB_020350]). The KI cassettes were composed of three separate fragments: (i) the gene of interest (various sizes) with its upstream flanking region (∼1 kb) and a terminator (between 150 and 200 bp), (ii) a zeocin resistance marker (*ble*) (∼2.1 kb), and (iii) the downstream flanking region (∼1 kb) of the gene of interest (see Fig. S1 in the supplemental material). A 2-step fusion PCR methodology was used to generate the above-described fragments in the 1st-step PCR (see reference 75). The sequences of the primers used are shown in Table S1. The genes with their upstream flanking sequences were amplified from A. fumigatus genomic DNA (A1160p^+^) using the primer set P1-KIP2. Downstream flanking regions were amplified with primers KIP3 and P4. The *ble* marker was amplified with primer set bleF-bleR (34). These primers contained common linker sequences complementary to the linker sequences in primers KIP2 and KIP3, respectively, allowing the amplification of a single *ble* marker for all 5 mutants. Following the 1st-step PCR, all fragments were analyzed on a 1% agarose gel and purified using a PCR purification kit (upstream flanking regions along with the genes and the downstream flanking sequences) or gel purified (the *ble* marker) (Qiagen). Subsequently, 2 μl of each fragment was used in the 2nd-step fusion PCR with primers P5 and P6 to generated final KI cassettes as described previously (74). The amplified fusion PCR products were analyzed on a 1% agarose gel before transformation. KI cassettes were transformed into the previously generated knockout mutants to complement for the gene loss using a transformation method described previously (74). Transformants were selected on Sabouraud medium containing 150 μg/ml zeocin (Invitrogen). The correct integration of the complementation cassette for the Δ*ccrA*, Δ*sltA*, and Δ*zipD* mutants was confirmed by PCR using primers P1 and bleR, primers P4 and bleF, and LongAmp *Taq* polymerase (New England BioLabs [NEB]) under the following conditions: 94°C for 2 min followed by 35 cycles of 94°C for 20 s, 58°C for 20 s, and 65°C for 3 min and 1 cycle of 65°C for 5 min. The correct integration of the complementation cassette for the Δ*ffmA* mutant was confirmed by the amplification of a 3.8-kb fragment with primers 1G9P1 and 1G9P4.

10.1128/mBio.00863-21.1 FIG S1 Schematic representation of the gene complementation strategy. (a) A fusion PCR approach was used to construct the complementation cassette. The upstream flank (Up flank) and the gene of interest (gene), along with ∼200 bp of the terminator (T), are amplified using locus-specific primers, designated P1 and KIP2, and the downstream flank (Down flank) is amplified using primers KIP3 and P4, while the zeocin-selectable marker (*ble*) is amplified using primers bleF and bleR. (b) Exemplar data for the amplification of the gene-specific fragments of 1D4 (4,067 bp [UG] and 932 bp [D]) and 2C7 (4,128 bp [UG] and 936 bp [D]). PCR-mediated fusion of the fragments is facilitated by the use of the nested primers P5 and P6. Amplification of the complementation cassette is shown for 1D4 (7,014 bp) and 2C7 (7,079 bp). Confirmation of the integration of the complementation cassette into the correct locus was performed by amplification with primers P1 and bleR (2C lane 1) (4,067 bp [1D4] and 4,128 bp [2C7]) and primers P4 and bleF (2C lane 2) (2,969 bp [1D4] and 2,998 bp [2C7]). Download FIG S1, PDF file, 0.4 MB. Copyright © 2021 Liu et al. 2021 Liu et al. https://creativecommons.org/licenses/by/4.0/ This content is distributed under the terms of the Creative Commons Attribution 4.0 International license.

10.1128/mBio.00863-21.9 TABLE S1 Complementation primers. The linker is italicized. Download Table S1, DOCX file, 0.01 MB. Copyright © 2021 Liu et al. 2021 Liu et al. https://creativecommons.org/licenses/by/4.0/ This content is distributed under the terms of the Creative Commons Attribution 4.0 International license.

Complementation for 1G9 (*ffmA* [AFUB_026340]) was carried out using the markerless CRISPR-Cas9 transformation protocol as described previously (76) (Fig. S2). The gene complementation cassette was generated by amplification of the coding sequence and ∼1-kb flanking regions for *ffmA* using primers 1G9r_for and 1G9r_rev from A1160p^+^ genomic DNA. Two CRISPR RNAs (IDT), Hph_48 and Hph_2661, that make a functional guide RNA (gRNA) when combined with trans-activating CRISPR RNA (IDT) were used to direct Cas9 to cleave the *hph* cassette in *ffmA*. Selection for the integration of the cassette was performed by selecting vigorously growing strains from the background of the slow-growing *ffmA* strain.

10.1128/mBio.00863-21.2 FIG S2 Reconstitution of *ffmA* via CRISPR-Cas9-mediated transformation. *ffmA* was reconstituted by using two crRNAs targeting the ends of the hygromycin selection cassette (indicated in black), replacing it with the *ffmA* gene directly. Five transformants were selected and PCR validated. As *ffmA* has a severe growth defect, reconstitution of the A1160p^+^ morphology was used as an indication of successful reconstitution. Download FIG S2, PDF file, 1.4 MB. Copyright © 2021 Liu et al. 2021 Liu et al. https://creativecommons.org/licenses/by/4.0/ This content is distributed under the terms of the Creative Commons Attribution 4.0 International license.

### Growth and conidiation.

Conidia of mutant and parental strains were obtained from mycelial cultures incubated on malt agar slants (2% [wt/vol] malt, 2% agar) at 37°C overnight, followed by room temperature for 3 to 7 days; harvested with a 0.05% (vol/vol) Tween 20 aqueous solution; and filtered through a 40-μm nylon cell strainer (BD Falcon). To analyze the sensitivity of strains to a variety of chemical perturbations, the appropriate amount of conidia was inoculated into buffered minimal medium (MM) (1% glucose, 0.092% ammonium tartrate, 0.052% KCl, 0.052% MgSO_4_ · 7H_2_O, 0.152% KH_2_PO_4_, 1 ml/liter trace element solution, 3.45% morpholinepropanesulfonic acid [MOPS] [pH 7.0]) or buffered RPMI medium (1.03% RPMI 1640 medium powder from Sigma [catalog no. R7755], 0.03% l-glutamine, 3.45% MOPS [pH 7.0]) with various concentrations of different drugs according the requirements of individual experiments (77, 78). Susceptibility to CR was assessed using a 2-fold dilution series (10 to 1,000 μg/ml) in MM solid medium (2% agar). Two drug concentrations, 50 μg/ml and 300 μg/ml, allowed the selection of sensitive and resistant mutants, respectively. To test the sensitivity of mutants in the presence of iron stresses, buffered MM agar medium was supplemented with 0.2 mM the iron-specific chelator bathophenanthrolinedisulfonic acid (BPS) or 5 mM FeSO_4_ (75). Serial 10-fold dilutions of conidia ranging from 10^6^ to 10^3^ cells in a volume of 5 μl were spotted onto the buffered MM agar plates. The plates were incubated at 37°C for 48 h and then imaged to determine the relative sizes of the colonies.

### Quantification of CR in liquid medium.

To analyze the ability of the strains to bind CR and remove it from the culture medium, the conidia of the wild type and mutants were inoculated in buffered MM with CR (100 μg/ml) and incubated with shaking (150 rpm) at 37°C. The concentration of CR was quantified at different times of growth by its absorbance at 500 nm after establishing a standard curve according to methods described previously (79). The half-concentration of CR in the medium was determined by plotting ln(*I*/*I*_0_) against time according to the equation ln(*I*/*I*_0_) = −*kt*, where *I*_0_ and *I* represent the initial and residual CR concentrations, respectively. The equation *t*_1/2_ = ln 2/*k* was used, where *t*_1/2_ is the time when the concentration in the medium reaches half of the initial CR concentration and *k* is the apparent elimination constant. In all cases, the first-order equation provided a satisfactory fit for the data (*r* > 0.9).

### Analysis of the cell wall monosaccharide components.

Analyses of the cell wall monosaccharide components were undertaken using methods described previously (78, 80). In summary, mycelium was harvested after 16 h of growth and disrupted with 0.5-mm glass beads in 0.2 M Tris-HCl buffer (pH 8.0). The disrupted mycelial suspension was centrifuged (4,500 × *g* for 10 min). The cell wall pellet was washed three times with distilled water and boiled in a 50 mM Tris-HCl buffer (pH 7.5) containing 50 mM EDTA, 2% SDS, and 40 mM β-mercaptoethanol for 15 min, twice. Total hexoses in the cell wall were quantified by the phenol sulfuric procedure using glucose as a standard. Neutral hexoses were analyzed by gas-liquid chromatography (GLC) as alditol acetates obtained after hydrolysis (4 N trifluoroacetic acid at 100°C for 6 h), reduction, and peracetylation. Derivatized monosaccharides were separated and quantified on a DB5 capillary column (25 m by 0.32 mm) using a Delsi 200 apparatus (carrier gas, 70 kPa helium; temperature program, 120°C to 180°C at 2°C/min and 180°C to 240°C at 4°C/min). After hydrolysis by 6 N HCl (100°C for 6 h), the *N*-acetylhexosamine in the cell wall was analyzed and quantified by high-performance anion-exchange chromatography (HPAEC) with a pulsed electrochemical detector and an anion-exchange column (CarboPAC PA-1, 4.6 by 250 mm; Dionex) using 18 mM NaOH as the mobile phase at a flow rate of 1 ml/min, and *N*-glucosamine and *N*-galactosamine were used as the standards.

### Analysis of the cell wall permeability of mutants with liposomes.

Liposomes labeled with rhodamine were used to analyze the cell wall permeability of mutants. Liposome formulations made of POPC (1-palmitoyl-2-oleoyl-*sn*-glycero-3-phosphocholine) and 18:1 Liss Rhod PE [1,2-dioleoyl-*sn*-glycero-3-phosphoethanolamine-*N*-(lissamine rhodamine B sulfonyl)] at 99:1 molar proportions were prepared according to the method of Bangham et al., followed by the extrusion of vesicle suspensions (81). Briefly, lipids (from Avanti Polar Lipids, Alabaster, AL, USA) were dispersed in chloroform at a 10 mM concentration. Organic lipid solutions were evaporated for 3 h at 60°C under reduced pressure, and the resulting dry lipid films were hydrated with phosphate-buffered saline (PBS) buffer and sonicated for a few minutes. The obtained liposome suspensions were then extruded 15 times successively through 200- and 50-nm-pore-diameter polycarbonate membranes at 40°C (Mini extruder; Avanti Polar Lipids, Alabaster, AL). The diameter of liposomes (size in nanometers and polydispersity index [PDI]) was measured by diffusion light scattering (DLS) using a Zeta-sizer (Nano ZS90; Malvern) after dilution of the suspension in buffer. The size of liposomes was 70 nm with a polydispersity index of <0.2. Suspensions of 5 × 10^6^ conidia/ml of the wild type and mutants were inoculated in buffered MM and incubated at 37°C for 14 to 16 h. The germinating conidia were collected by centrifugation at 4,500 × *g* for 10 min, resuspended in HEPES buffer (10 mM, pH 7.0) with 20 μl liposomes labeled with 1% rhodamine PE, and incubated at 37°C for 3 h. Fluorescence and differential interference contrast (DIC) images were captured with a Zeiss Axio Imager A1 microscope (Zeiss, Jena, Germany). The fluorescence intensity was analyzed by ImageJ 1.8.0 (82). The fluorescence intensity was quantified by ImageJ 1.8.0 from an average of 9 pictures.

### Western blot analysis to analyze the phosphorylation of MpkA.

Conidia of wild-type Δ*ku80* and the Δ*zipD*, Δ*ffmA*, Δ*ccrA*, and Δ*sltA* mutant strains were inoculated in buffered MM medium without or with CR (100 μg/ml) and incubated at 37°C at 150 rpm. Mycelium was harvested at 12 h and disrupted with 0.5-mm glass beads in lysis buffer containing 10% (vol/vol) glycerol, 50 mM Tris-HCl (pH 7.5), 1% (vol/vol) Triton X-100, 150 mM NaCl, 0.1% (wt/vol) SDS, 5 mM EDTA, 50 mM sodium fluoride, 5 mM sodium pyrophosphate, 50 mM beta-glycerophosphate, 5 mM sodium orthovanadate, 1 mM phenylmethylsulfonyl fluoride (PMSF), and 1× protease inhibitor (Roche Applied Science, Germany). Extracts were centrifuged at 14,000 × *g* for 15 min at 4°C. The phosphorylation levels of MpkA were analyzed by Western blotting (83). The phosphorylation state and total MpkA were examined using anti-phospho-p44/42 MAPK and anti-p44/42 MAPK antibodies (catalog no. 9101 and 4370; Cell Signaling Technologies) according to the manufacturer’s instructions. Signal intensities were quantified using ImageJ software by dividing the intensity of MpkA-P by that of MpkA.

### Transcriptomic analysis.

For RNA-seq experiments, two types of culture conditions were undertaken. Strains were grown in buffered MM for 16 h following the addition of CR to one set of flasks at the desired concentrations, i.e., 300 μg/ml for the resistant mutants (Δ*ccrA*, Δ*sltA*, and the wild type) and 50 μg/ml for the sensitive mutants (Δ*zipD*, Δ*ffmA*, and the wild type). The strains were incubated for an additional 4 h before collecting the mycelia and processing them for RNA extraction according to the phenol-chloroform method. In another experiment, the CR-resistant Δ*sltA* and Δ*zipD* mutants were incubated in buffered MM in the presence of 300 μg/ml CR for 4 days. RNA extraction was carried out using a previously described phenol-chloroform-based method (84). Total RNAs from three independent replicates were checked on the Bioanalyzer system (Agilent) for their quality and integrity. Polyadenylated RNA purification and indexed libraries were performed using the TruSeq stranded mRNA sample preparation kit according to the manufacturer’s instructions (Illumina). Libraries were checked for quality on Bioanalyzer DNA chips (Agilent). More precise and accurate quantification was performed with the fluorescence-based quantitation Qubit dsDNA HS assay kit (Thermo Fisher). Fifty-one-base-pair single-read sequences were generated on the Hiseq2500 sequencer according to the manufacturer’s instructions (Illumina). The level of sample multiplexing was 6 per lane. Reads were cleaned of adapter sequences and low-quality sequences using an in-house program (https://github.com/baj12/clean_ngs). Only sequences of at least 25 nucleotides (nt) in length were considered for further analysis. HISAT2 version 2.1.0, with default parameters, was used for alignment on the reference genome (Aspergillus fumigatus A1163 from Ensembl release 47). Genes were counted using featureCounts version 1.6.4. The change of gene expression levels was calculated as the fold change of the gene reads for the WT under low- or high-CR conditions to those in medium without CR. Additionally, reads from mutant strains exposed to CR were compared to those from mutant strains without CR. Count data were analyzed separately for “low,” “high,” and parental and mutant samples using DESeq2 version 2.11.40.6 (85). The normalization and dispersion estimations were performed with DESeq2 using the default parameters, and statistical tests for differential expression were performed by applying the independent filtering algorithm. A generalized linear model was set in order to test for differential expression between the different biological conditions using R (R Project for Statistical Computing [https://www.r-project.org]). For each pairwise comparison, raw *P* values were adjusted for multiple testing according to methods described previously (86), and genes with an adjusted *P* value (*P*_adj_) of <0.05 were considered differentially expressed. Gene ontology analysis was performed using FungiFun2 and DAVID (87, 88). Heat maps were made in R using pheatmap (89).

### Data availability.

Our transcriptome data were submitted to the GEO under reference accession no. GSE140840 (https://www.ncbi.nlm.nih.gov/geo/query/acc.cgi). Output from DESeq2 on our transcriptome data can be found at https://doi.org/10.48420/14252756 for RNA-seq data after 4 h of incubation of the parental and sensitive and resistant mutants with CR, https://doi.org/10.48420/14252768 for RNA-seq data showing the expression of genes involved in sterol metabolism and synthesis of secondary metabolite metabolisms after 4 h of incubation of the parental and sensitive and resistant mutants with CR, and https://doi.org/10.48420/14252759 for RNA-seq data after 4 days of incubation of the parental and CR-resistant mutants with high concentrations of CR.

## ACKNOWLEDGMENTS

This work was partly funded by grants from Agence Nationale de la Recherche (ANR-16-CE92-0039) and Fondation pour la Recherche Médicale (DEQ20150331722) to J.-P.L., from Wellcome (219551/Z/19/Z) to N.V.R., and from Wellcome (208396/Z/17/Z) to M.B.
