# Supplementary material for: Functional Genomic and Biochemical Analysis Reveals Pleiotropic Effect of Congo Red on Aspergillus fumigatus
Source: mBio. 2021 May 18;12(3):e00863-21. doi: 10.1128/mBio.00863-21 (PMC8262895; doi:10.1128/mBio.00863-21)
Supplement: FIG S3 [file mbio.00863-21-sf003.pdf]

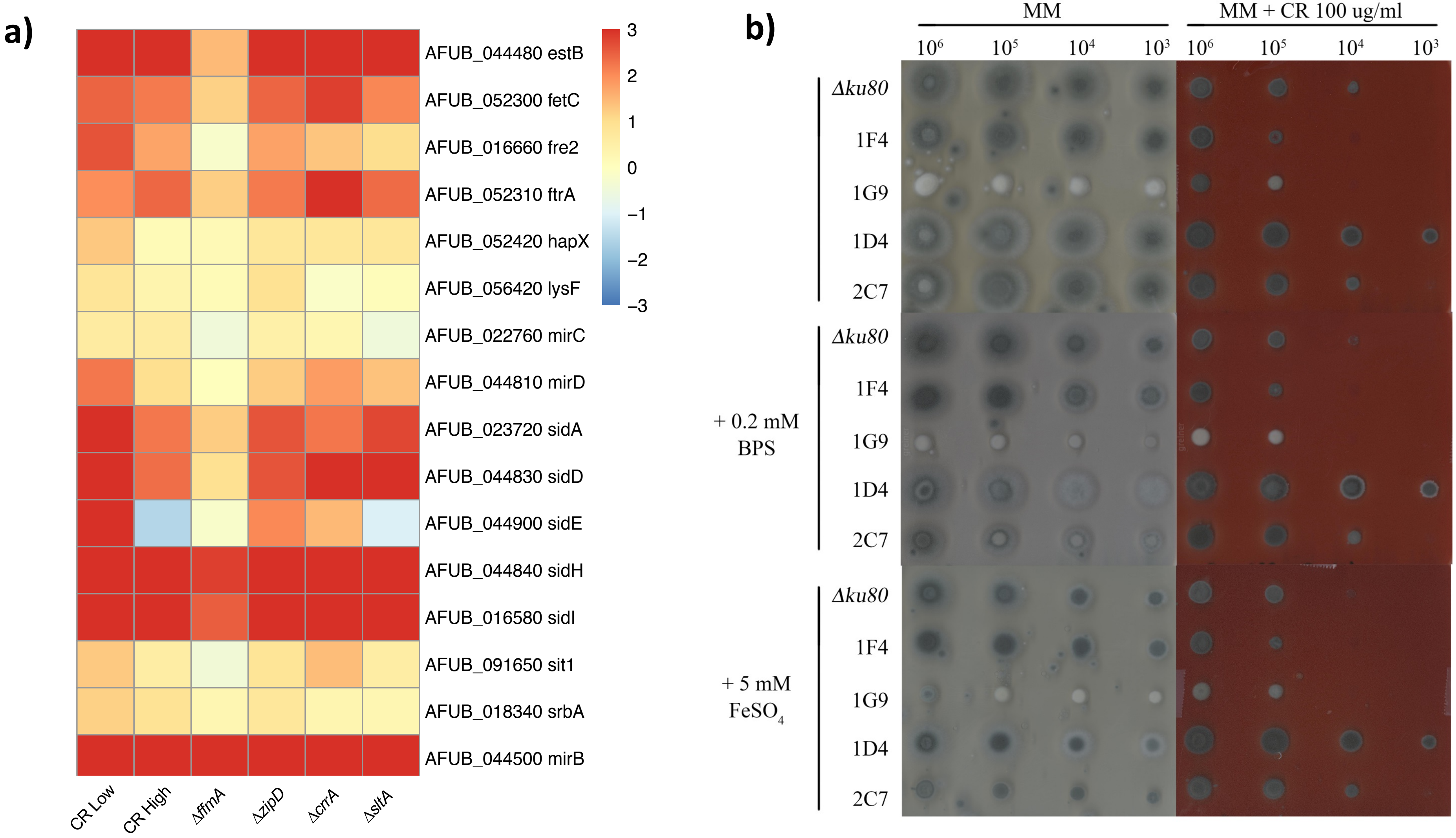

Figure S3 Iron is not involved in the degree of resistance of strains to CR (a) Heat map of the genes involved in iron metabolism in the parental strain and the 4 TF mutants. (b) In the presence of iron-starvation induced by the specific chelator BPS or after supplementation with a high concentration of iron ($FeSO_4$), the sensitivity of strains to CR was not modified.
