# Supplementary material for: Functional Genomic and Biochemical Analysis Reveals Pleiotropic Effect of Congo Red on Aspergillus fumigatus
Source: mBio. 2021 May 18;12(3):e00863-21. doi: 10.1128/mBio.00863-21 (PMC8262895; doi:10.1128/mBio.00863-21)
Supplement: FIG S4 [file mbio.00863-21-sf004.pdf]

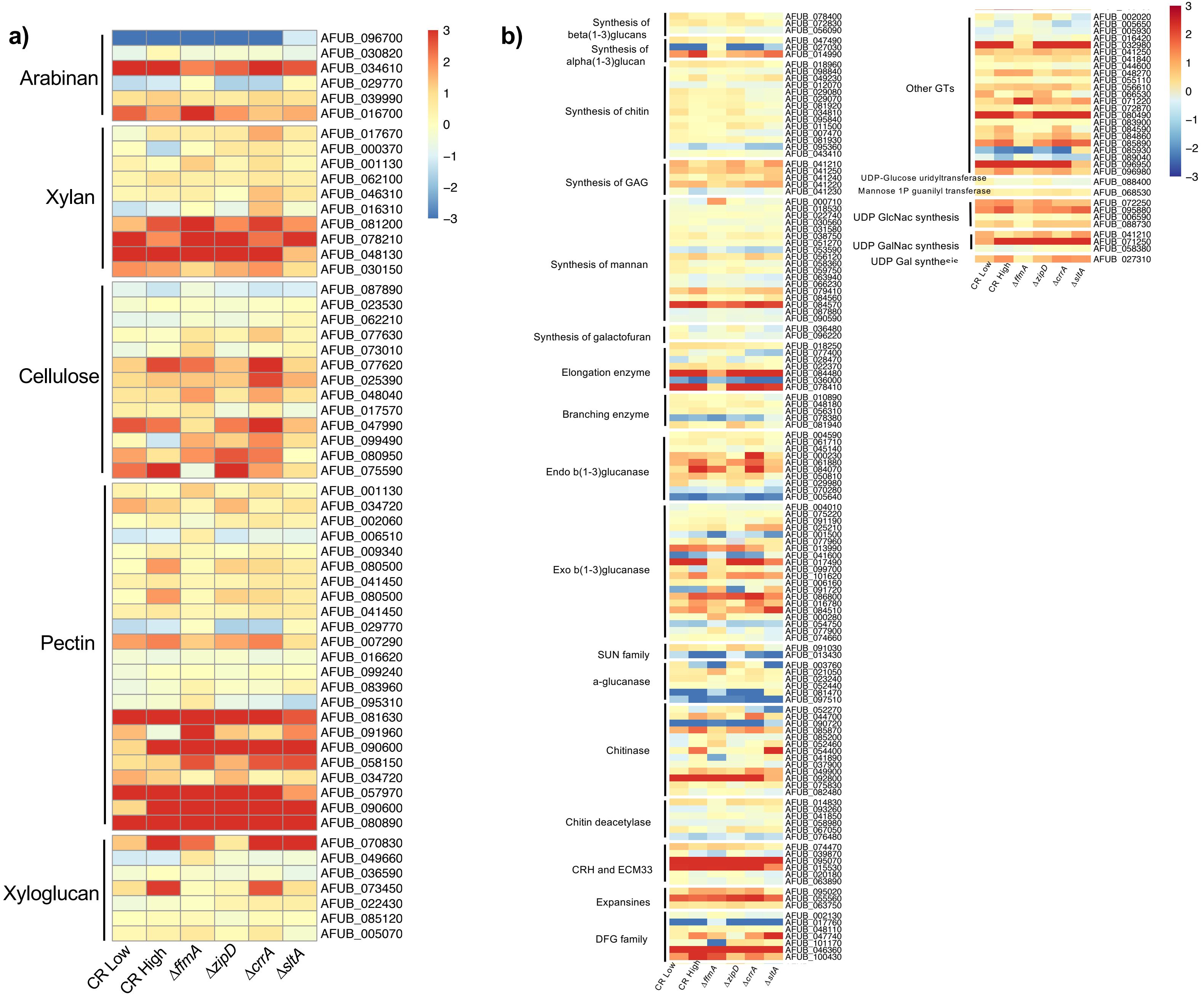

Figure S4 Glycosylhydrolase and cell wall genes. Expression of the glycosylhydrolase genes involved in the degradation of plant decaying material (a) and cell wall associated genes (b) by the parental and TF mutants.
