# Supplementary material for: Functional Genomic and Biochemical Analysis Reveals Pleiotropic Effect of Congo Red on Aspergillus fumigatus
Source: mBio. 2021 May 18;12(3):e00863-21. doi: 10.1128/mBio.00863-21 (PMC8262895; doi:10.1128/mBio.00863-21)
Supplement: FIG S5 [file mbio.00863-21-sf005.pdf]

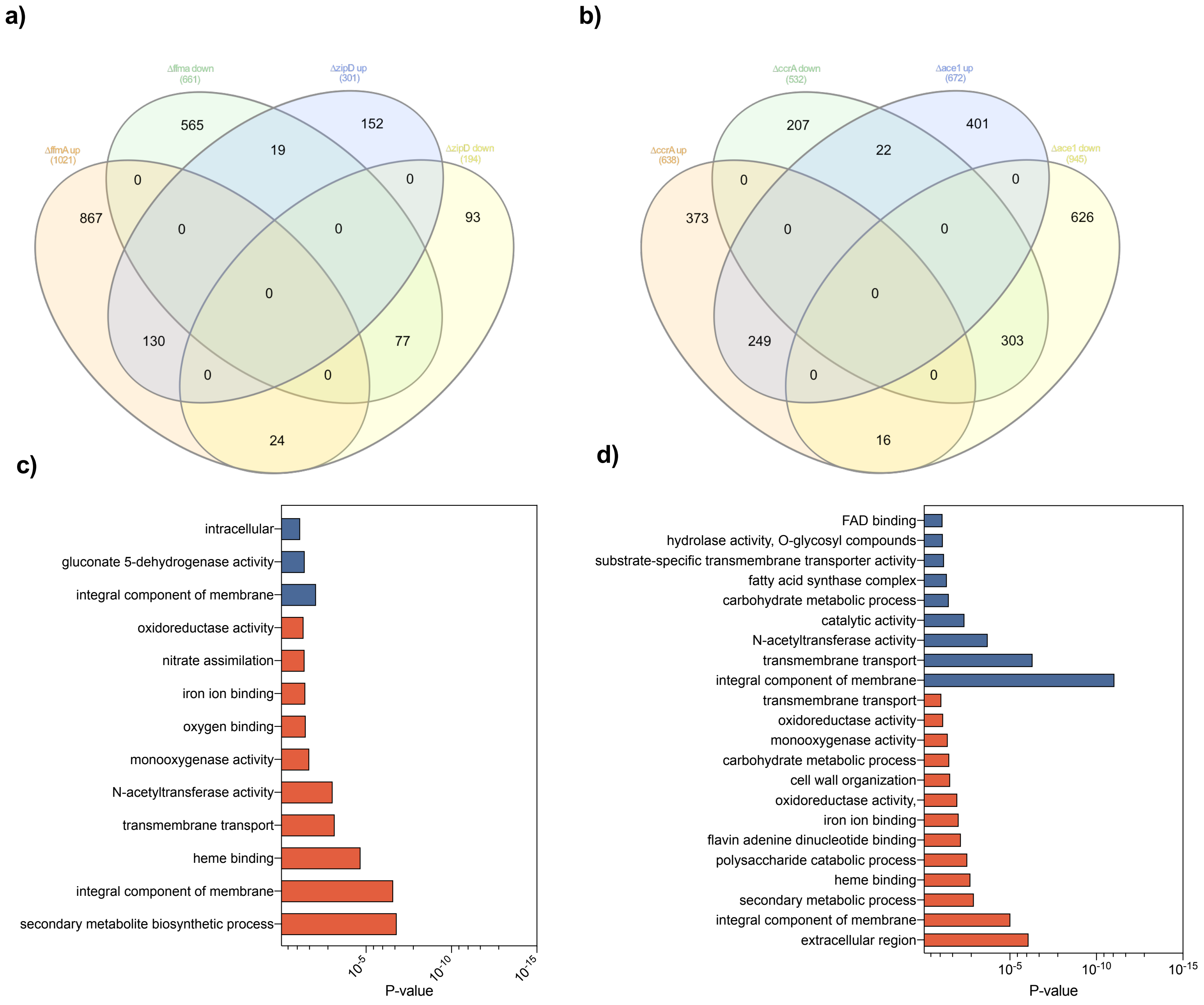

Figure S5 RNA-seq analysis of TF mutants. A and b) Venn diagrams to identify genes commonly differentially regulated in sensitive (a) and resistant (b) isolates. (c and d) gene ontology analysis by FungiFun2 and DAVID to identify functional categories enriched within commonly up- or downregulated genes in sensitive (c) and resistant (d) isolates.
