# Supplementary material for: Functional Genomic and Biochemical Analysis Reveals Pleiotropic Effect of Congo Red on Aspergillus fumigatus
Source: mBio. 2021 May 18;12(3):e00863-21. doi: 10.1128/mBio.00863-21 (PMC8262895; doi:10.1128/mBio.00863-21)
Supplement: FIG S6 [file mbio.00863-21-sf006.pdf]

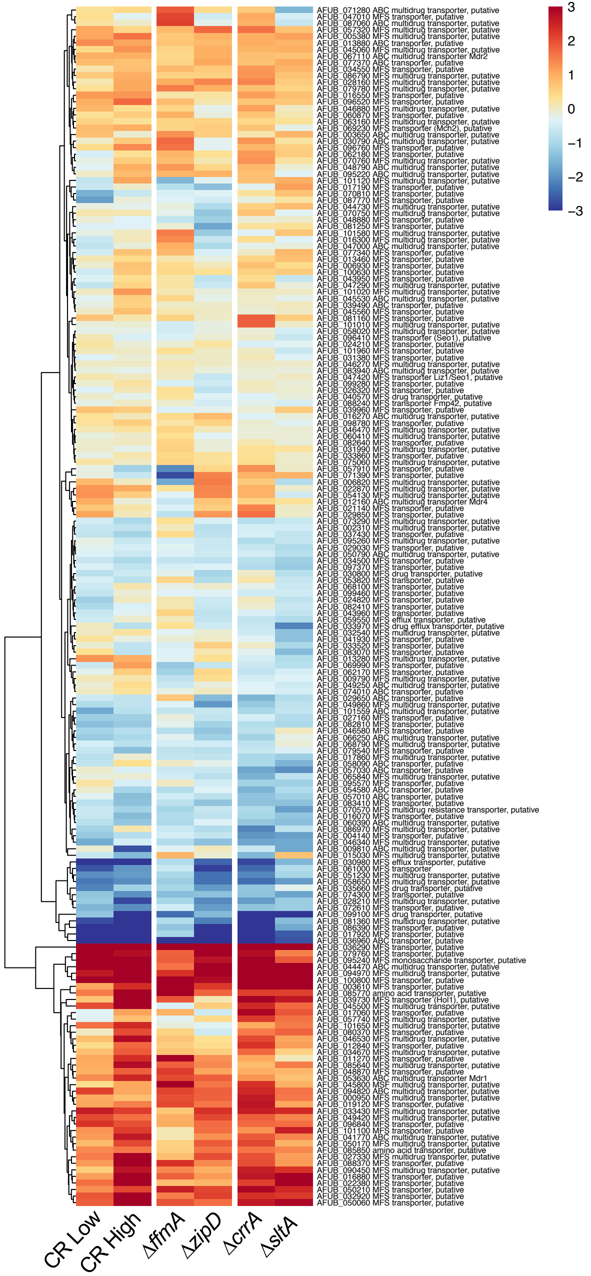

Figure S6 Differentially regulated *A. fumigatus* transporters upon Congo Red exposure. Heatmap of predicted MFS and ABC transporters (2> or <-2 differentially regulated in at least one conditions) in *A. fumigatus* in Δ*ffmA*, Δ*ace1*, Δ*zipD*, Δ*crrA*.
