# Supplementary material for: Functional Genomic and Biochemical Analysis Reveals Pleiotropic Effect of Congo Red on Aspergillus fumigatus
Source: mBio. 2021 May 18;12(3):e00863-21. doi: 10.1128/mBio.00863-21 (PMC8262895; doi:10.1128/mBio.00863-21)
Supplement: FIG S7 [file mbio.00863-21-sf007.pdf]

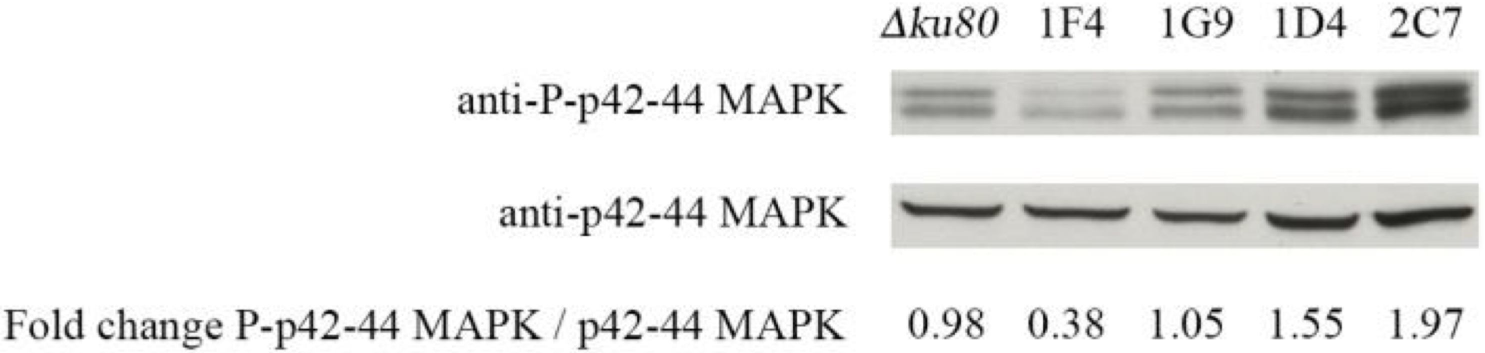

Figure S7. Immunoblot analysis of the MpkA phosphorylation of wild type and mutants in the medium with CR. Anti-phosphop44/42 MAPK or Anti-44/42 MAPK antibodies directed against phosphorylated MpkA and total MpkA were used to detect the phosphorylation of MpkA and total MpkA respectively. Signal intensities were quantified using the ImageJ software by dividing the intensity of MpkA-P/MpkA.
