# Supplementary material for: Functional Genomic and Biochemical Analysis Reveals Pleiotropic Effect of Congo Red on Aspergillus fumigatus
Source: mBio. 2021 May 18;12(3):e00863-21. doi: 10.1128/mBio.00863-21 (PMC8262895; doi:10.1128/mBio.00863-21)
Supplement: FIG S8 [file mbio.00863-21-sf008.pdf]

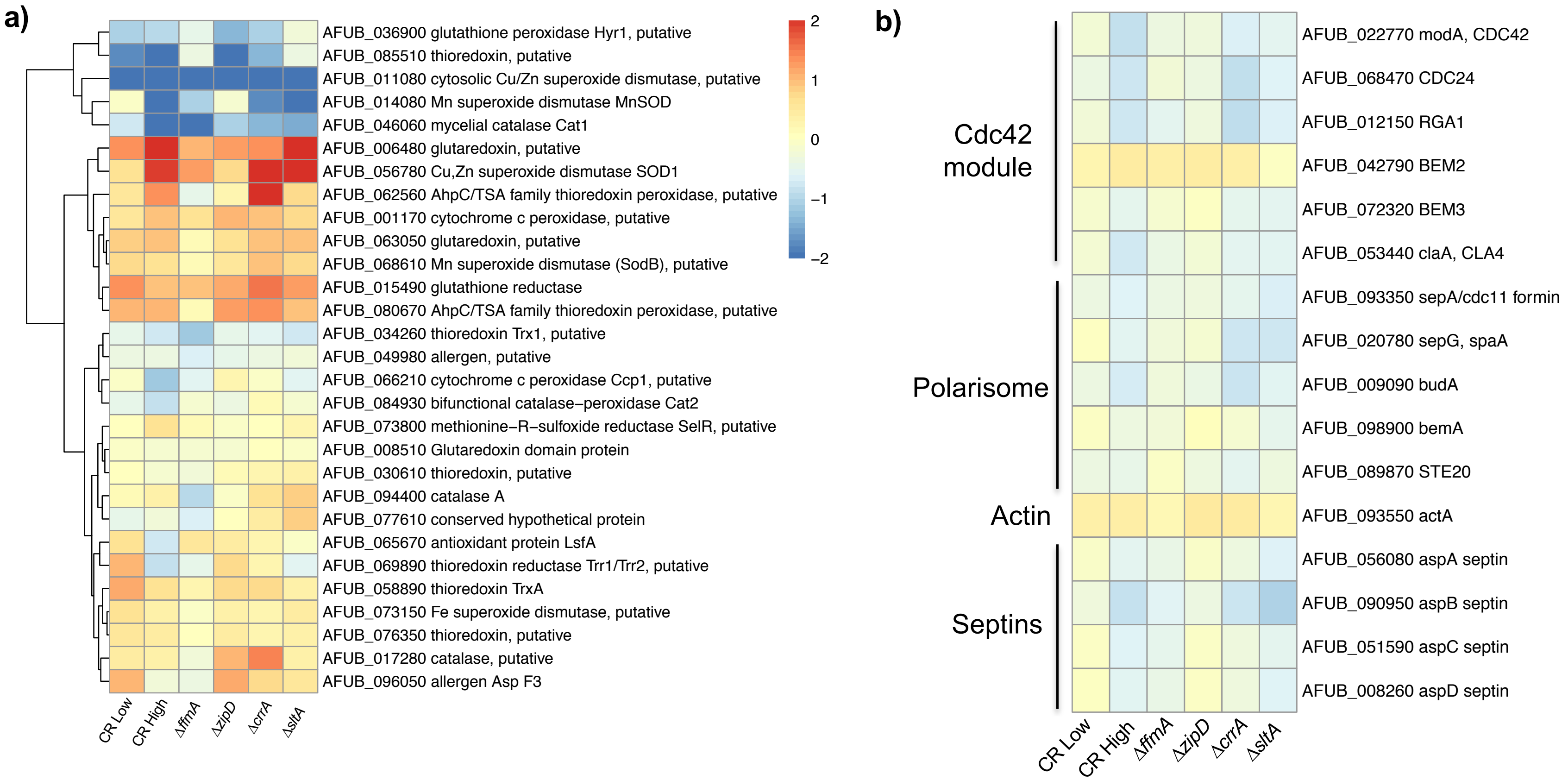

Figure S8 ROS and polarisation genes. Expression of the genes involved in the resistance Of reactive oxidant stress (a) and genes associated with polarisation of filamentous growth (b).
