# Supplementary material for: Functional Genomic and Biochemical Analysis Reveals Pleiotropic Effect of Congo Red on Aspergillus fumigatus
Source: mBio. 2021 May 18;12(3):e00863-21. doi: 10.1128/mBio.00863-21 (PMC8262895; doi:10.1128/mBio.00863-21)
Supplement: FIG S1 [file mbio.00863-21-sf001.pdf]

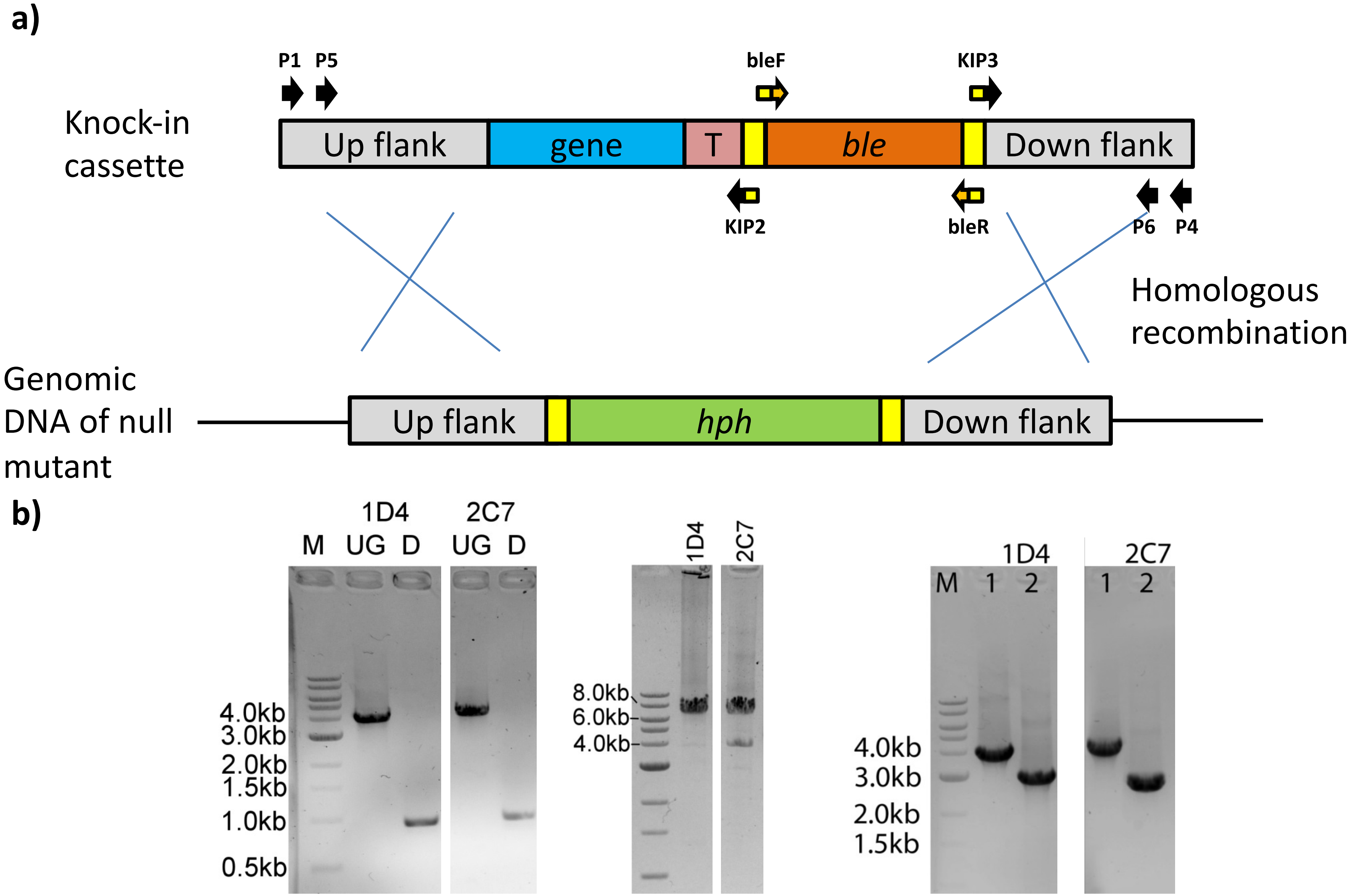

Figure S1 Schematic representation of the gene complementation strategy. a) A fusion PCR approach was used to construct the complementation cassette. The upstream flank (Up flank) and gene of interest (gene), along with c. 200bp of the terminator (T) is amplified using locus specific primers designated P1 and KIP2, the downstream flank (Down flank) is amplified using primers KIP3 and P4 while the zeocin selectable marker (ble) is amplified using primers bleF and bleR. b) Exemplar data for amplification of the gene specific fragments 1D4 (4067bp [UG] and 932 bp [D]) and 2C7 (4128bp [UG] and 936bp [D]). PCR mediated fusion of the fragments is facilitated by the use of the nested primers P5 and P6. Amplification of the complementation cassette is shown for 1D4 (7014bp) and 2C7 (7079). Confirmation of integration of the complementation cassette into the correct locus was confirmed by amplification with primers P1 and bleR (2C lane 1; 4067bp [1D4], 4128bp [2C7]) and primers P4 and bleF (2C lane 2; 2969bp [1D4], 2998bp [2C7]).
