# Supplementary material for: Functional Genomic and Biochemical Analysis Reveals Pleiotropic Effect of Congo Red on Aspergillus fumigatus
Source: mBio. 2021 May 18;12(3):e00863-21. doi: 10.1128/mBio.00863-21 (PMC8262895; doi:10.1128/mBio.00863-21)
Supplement: TABLE S1 [file mbio.00863-21-st001.docx]

**Supplementary table 1. Complementation Primers (**Linker is italicised)

bleF – 5’-***CCGGCTCGGTAACAGAA***CTAACGGCGTAACCAAAAGTCAC-3’

bleR - 5’-***GTTGGAGCATATCGTTCAGAGC***TCTTGACGACCGTTGATCTG-3’

**AFUB_014780 – 1D4**

1D4P1 – 5’-CTGGCTCTGTTCTTTCGACC-3’

1D4KIP2 – 5’-***TAGTTCTGTTACCGAGCCGG***CGACAAGATCCATTCGGAAG-3’

1D4KIP3 – 5’-***GCTCTGAACGATATGCTCCAAC***ACATCCGGGACGAGATAATG-3’

1D4P4 – 5’-CCACAAAGCTCTCAACCCTC-3’

1D4P5 – 5’-TCCTTAAAGCGCACAGTCATT-3’

1D4P6 – 5’-TGAGTCACCGCATGGTATGT-3’

**AFUB_041100 – 2C7**

2C7P1 – GAGGGCCAGAGAGGGTGGAAC

2C7KIP2 – ***TAGTTCTGTTACCGAGCCGG***TAACAGAACATGCCGCACTC

2C7KIP3 – ***GCTCTGAACGATATGCTCCAAC***CCCCAAGTTGACGTTGATCT

2C7P4 – CGTTCAATCGATCCGTTGAG

2C7P5 – CTGATATCTTTATCCCAGCC

2C7P6 – GGATCTATCACTTCGATCCG

**AFUB_020350 – 1F4**

1F4P1 - CCCCAGCTACCACACTTCTT

1F4KIP2 - ***TAGTTCTGTTACCGAGCCGG***TCGATGTAAGCCGAGATTCA

1F4KIP3 - ***GCTCTGAACGATATGCTCCAAC***AGTCGCAGAACAGCCACTTT

1F4P4 - TCTTGCCAGGGAGAGAGAAA

1F4P5 - TTCCCTCCCCTCTTTTCTTC

1F4P6 – ATGAAACCTCACTGGCTGCT

**AFUB_026340 – 1G9**

1F4P1 - TTCTGTTTTTCTCGCACGTC

1F4P4 - TTCTTTCATTCCGTGTTCCC

1G9r_for: ACCGTACGTACCTTGGTGCT

1G9r_rev: CAACCCGATCGTAAGAAGGA

crRNA to target hph cassette

Hph_48 AAGCUGUAAGGAUUUCGGCACGG

Hph_2661 AGCAGAGCUAUUUUCAUUUUCGG
