# Supplementary material for: Functional Genomic and Biochemical Analysis Reveals Pleiotropic Effect of Congo Red on Aspergillus fumigatus
Source: mBio. 2021 May 18;12(3):e00863-21. doi: 10.1128/mBio.00863-21 (PMC8262895; doi:10.1128/mBio.00863-21)
Supplement: FIG S2 [file mbio.00863-21-sf002.pdf]

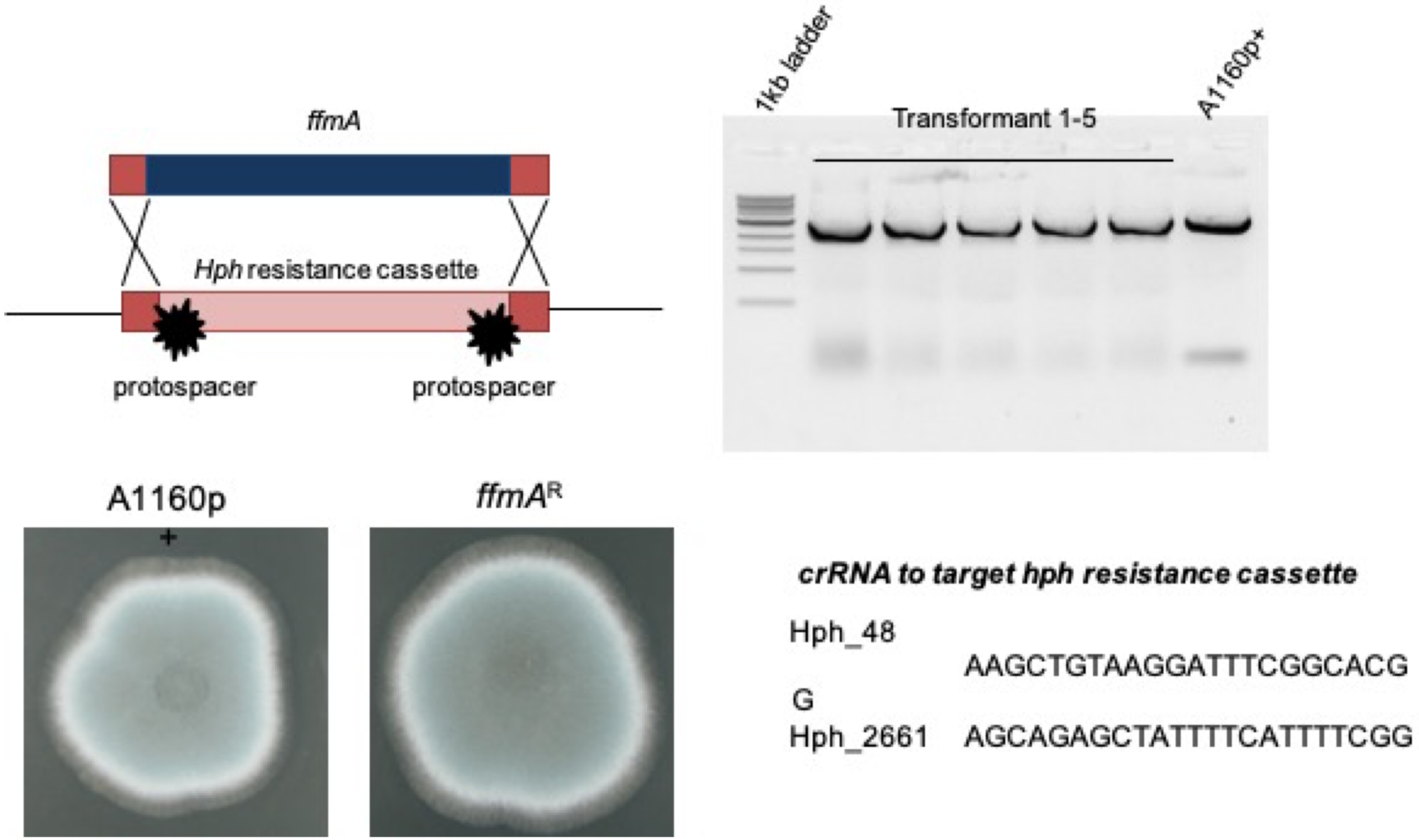

Figure S2 Reconstitution of ffmA via CRISPR-Cas9 mediated transformation. *ffmA* was reconstituted by using two crRNAs targeting the ends of the hygromycin selection cassette (indicated in black), replacing it with the *ffmA* gene directly. Five transformants were selected and PCR validated. As *ffmA* has a severe growth defect, reconstitution of the A1160p+ morphology was used as an indication of successful reconstitution.
